# Estimation of the Controlled Release of Antioxidants from β-Cyclodextrin/Chamomile (*Matricaria chamomilla* L.) or Milk Thistle (*Silybum marianum* L.), Asteraceae, Hydrophilic Extract Complexes through the Fast and Cheap Spectrophotometric Technique

**DOI:** 10.3390/plants12122352

**Published:** 2023-06-17

**Authors:** Adina Horablaga, Alina Şibu (Ciobanu), Corina Iuliana Megyesi, Dina Gligor (Pane), Gabriel Stelian Bujancă, Ariana Bianca Velciov, Florica Emilia Morariu, Daniel Ioan Hădărugă, Corina Dana Mişcă, Nicoleta Gabriela Hădărugă

**Affiliations:** 1 Department of Sustainable Development and Environmental Engineering, University of Life Sciences “King Mihai I” from Timişoara, Calea Aradului 119, 300645 Timişoara, Romania or adinahorablaga@usvt.ro; 2 Doctoral School “Engineering of Vegetable and Animal Resources”, University of Life Sciences “King Mihai I” from Timişoara, Calea Aradului 119, 300645 Timişoara, Romania; ciobanualina1966@yahoo.com (A.Ş.); gligor_dina_bihor@yahoo.com (D.G.); daniel.hadaruga@upt.ro (D.I.H.); 3 Department of Food Science, University of Life Sciences “King Mihai I” from Timişoara, Calea Aradului 119, 300645 Timişoara, Romania; corina.megyesi@usvt.roarianavelciov@usvt.ro; 4 Department of Food Control, University of Life Sciences “King Mihai I” from Timişoara, Calea Aradului 119, 300645 Timişoara, Romania; gabrielbujanca@yahoo.com; 5 Department of Biotechnologies, University of Life Sciences “King Mihai I” from Timişoara, Calea Aradului 119, 300645 Timişoara, Romania; 6 Department of Applied Chemistry, Organic and Natural Compounds Engineering, Polytechnic University of Timişoara, Carol Telbisz 6, 300001 Timişoara, Romania

**Keywords:** β-cyclodextrin complexes, transdermal pharmaceutical formulations, controlled release, chamomile, *Matricaria chamomilla* L., milk thistle, *Silybum marianum* L., ethanolic extract, spectrophotometric analysis, Korsmeyer–Peppas model

## Abstract

This is the first study on the modeling of the controlled release of the estimated antioxidants (flavonoids or flavonolignans) from β-cyclodextrin (β-CD)/hydrophilic vegetable extract complexes and the modeling of transdermal pharmaceutical formulations based on these complexes using an overall estimation by the spectrophotometric method. The Korsmeyer–Peppas model was chosen for evaluating the release mechanisms. β-CD/chamomile (*Matricaria chamomilla* L., Asteraceae) ethanolic extract and β-CD/milk thistle (*Silybum marianum* L., Asteraceae) ethanolic extract complexes were obtained by the co-crystallization method with good recovering yields of 55–76%, slightly lower than for β-CD/silibinin or silymarin complexes (~87%). According to differential scanning calorimetry (DSC) and Karl Fischer water titration (KFT), the thermal stability of complexes is similar to β-CD hydrate while the hydration water content is lower, revealing the formation of molecular inclusion complexes. In the Korsmeyer–Peppas model, β-CD/*M. chamomilla* flower extract complexes reveal Case II transport mechanisms, while the corresponding complexes with leaf extracts indicate non-Fickian diffusion for the controlled release of antioxidants in ethanol 60 and 96%. The same non-Fickian diffusion was revealed by β-CD/*S. marianum* extract and β-CD/silibinin complexes. On the contrary, almost all model transdermal pharmaceutical formulations based on β-CD/*M. chamomilla* extract complexes and all those based on β-CD/*S. marianum* extract complexes revealed non-Fickian diffusion for the antioxidant release. These results indicate that *H*-bonding is mainly involved in the diffusion of antioxidants into a β-CD based matrix, while the controlled release of antioxidants in model formulations is mainly due to hydrophobic interactions. Results obtained in this study can be further used for studying the particular antioxidants (namely rutin or silibinin, quantified, for example, by liquid chromatographic techniques) for their transdermal transport and biological effects in innovatively designed pharmaceutical formulations that can be obtained using “green” methods and materials.

## 1. Introduction

*Matricaria chamomilla* L. (chamomile) and *Silybum marianum* L. (milk thistle) are two medicinal plant species that belong to the same botanical family, Asteraceae. They are important species of medicinal and aromatic plants (MAPs). The area cultivated with MAPs exceeding 200,000 ha in Europe [1].

Chamomile is one of the most cultivated MAPs in the world, with more than 20,000 ha [2]. Among various biological activities, chamomile flowers are used for their anti-inflammatory and antioxidant activities, for digestive, uterine muscle, menstrual, and postpartum disorders, as well as for anxiety, cancer prevention, cardiovascular diseases, diabetes, liver disorders, and neuralgic pains [3,4,5]. Chamomile is traditionally used as an infusion, decoction, vapor inhalation, compress, and tea [5]. On the other hand, chamomile essential oils, extracts, or cell suspension cultures are studied for their antioxidant and biological activities, which are mainly due to the presence of essential oils and/or antioxidant polyphenolic components [3,6,7,8,9,10]. Various extraction methods are used for obtaining essential oils or polyphenol-based extracts [4,11]. Essential oils can be extracted by classical hydrodistillation, steam distillation, solid-liquid extraction using hydrophobic solvents, or extraction using supercritical fluids [6,8,12,13,14]. The essential oil yields reach 1.77% for some *M. chamomilla* genotypes, but the average yield is in the range of 0.8–0.9% [12]. The main essential oil compounds are bisabolol oxides A and B (21.6–47.3% and up to 20.6%), bisabolone oxide A (up to 12.4%), as well as camazulene (<19.9%). Dicycloethers such as *cis*-tonghaosu have been determined in *M. chamomile* L. essential oils at 2.3–46% [12,13,14,15]. On the other hand, polyphenolic extracts are generally obtained by cold or hot extraction using methanol or ethanol as solvents (e.g., Soxhlet extraction), microwave-assisted extraction, ultrasound-assisted extraction, subcritical water extraction, or supercritical CO_2_ extraction [3,4,6,7,11]. The polyphenolic compounds of the hydrophilic *M. chamomile* L. extracts belong to the flavonoids and phenolic acid classes. Among flavonoids, quercetin and its glycosides, rutin, chrysin, apigenin, and luteolin 7-*O*-glucoside (1.07–1.97, 1.76–4.40, 3.52–11.69, and 4.70–9.17 mg/g dry extract, respectively) were the most concentrated in the 70% ethanol extracts [3,9,16]. The main phenolic acids were chlorogenic, *p*-coumaric, cafeic, and ferulic acids, with maximum contents up to 14.8% for *p*-coumaric acid [3]. They were also quantified as total phenol content (TPC) or total flavonoid content (TFC), with values of 117.3–151.5 mg chlorogenic acid equivalents (CAE)/g and 49.7–64.3 mg rutin equivalents (RE)/g, respectively [4]. The copper excess in the nutrient solutions used for *M. chamomile* L. cultivation can enhance the accumulation of some phenolic contents [17]. The overall antioxidant activities of *M. chamomilla* L. extracts were evaluated through various methods, including the DPPH· (2,2-diphenyl-1-pycrylhydrazyl radical) assay, ferrous ion chelating ability, reducing power, hydroxyl radical scavenging activity, or ABTS^+^· [2,2′-azino-bis(3-ethylbenzothiazoline-6-sulfonic acid radical cation)] assay [7,11].

*Silybum marianum* L. is widely used for its valuable hepatoprotective effect. This effect is mainly due to the presence of flavonolignans, which are polyphenols derived from flavonoids. A complex mixture of flavonolignans can be found in *S. marianum* extracts, including silibinins and silychristins. Silymarin is the standardized *S. marianum* fruit extract. It mainly contains the above-mentioned flavonolignans and up to 25% oil [18,19]. The extraction of bioactive flavonolignans is generally performed by classical methods using organic solvents, with some disadvantages related to selectivity and toxicity. On the other hand, supercritical fluid extraction is a green alternative for the separation of silymarin extracts [20]. The antioxidant activity of *S. marianum* extract is important. Both flavonolignans and flavonoids, such as silibinins, taxifolin, and quercetin, provide antioxidant effects. Among them, chlorogenic acids and tocopherols also have an impact [21,22,23]. The contents of various antioxidant compounds in European *S. marianum* genotypes were in the range of 3086–9499 mg/kg for silibinins, 126.5–395.3 mg/kg for chlorogenic acids, and 3.5–79.7 mg/kg for luteolin, with TFC values of 30–84 mg RE/100 g [24]. The total antioxidant activity of *S. marianum* extracts strongly depends on the plant’s development, growing conditions, and treatments, as well as the extraction procedure. The maximum antioxidant activity was observed in 80-day-old leaves and plants (60 and 65.43% by DPPH· assay, respectively) [25]. The antioxidant activity of the achene extracts of different *S. marianum* varieties was determined by various methods. The radical scavenging activity was in the range of 1763–5303 μmol TE/100 g by the DPPH· assay, 2965–14426 μmol TE/100 g by the ORAC assay, and 692–1664 μmol TE/100 g by the FRAP assay [24].

Polyphenols are easily oxidized in proper conditions, especially if they are not appropriately stabilized. Flavonoids such as quercetin and chrysin can be transformed to free radicals and further to quinone methides by various mechanisms, including hydrogen atom transfer (HAT) or single-electron transfer (SET) mechanisms. Metal ions are also involved in the antioxidant mechanism of polyphenols [26,27]. The modulation of the antioxidant activity of polyphenols and the corresponding extracts can be achieved by nanoencapsulation [28]. Among many encapsulation matrices, cyclodextrins (CDs) are appropriate natural compounds for molecular inclusion of the most known polyphenols, including flavonoids and flavonolignans [29,30]. They can be completely or partially encapsulated into the CD cavity, depending on the structural characteristics (hydrophobicity and dimensions) [31,32]. As a consequence, the reactivity of antioxidants is apparently reduced by CD nanoencapsulation, and a controlled release supramolecular system can be obtained [33,34,35]. Many studies deal with the CD complexation of “pure” antioxidant flavonoids and flavonolignans [34,35,36,37,38,39,40,41], but only a few of them are related to the CD nanoencapsulation of *M. chamomilla* or *S. marianum* extracts [42,43,44,45]. Some similar complexes were also obtained and studied in our laboratory. They were especially related to the CD encapsulation of *Compositae* essential oils [13,14,46].

In this study, a comparison between the thermal stability and controlled release by spectrophotometric evaluation of β-CD/chamomile (*Matricaria chamomilla* L.) or milk thistle (*Silybum marianum* L.), Asteraceae, hydrophillic extract complexes was performed. It mainly focuses on the applicative aspects regarding the β-CD complexation of the raw ethanolic extracts and the controlled release from the complexes and transdermal pharmaceutical formulations of the main antioxidant compounds (e.g., chrysin, rutin, and silibinins/silymarin, respectively) in various media.

## 2. Results and Discussion

### 2.1. Obtaining of Chamomile (Matricaria chamomilla L.) and Milk Thistle (Silybum marianum L.) Hydrophillic Extracts

**Figure 1:**
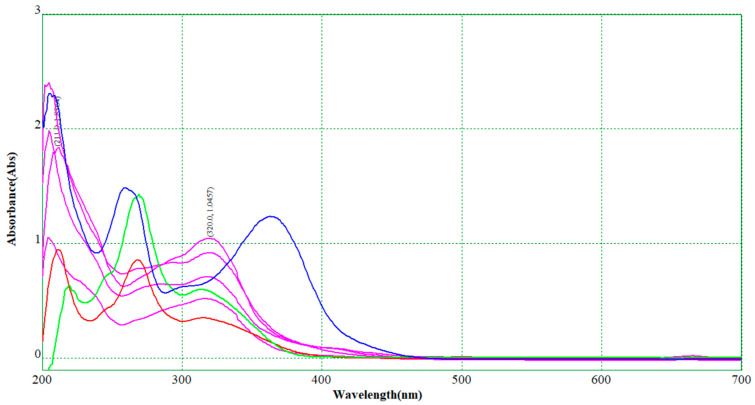
Superimposed UV–Vis spectra for *M. chamomilla* flower, leaf, root, and stem extracts (various dilutions, pink), standard rutin and chrysin solutions (38.0 μg/mL and 16.8 μg/mL, green and blue), respectively, and the mixture of rutin (0.76 μg/mL) and chrysin (6.72 μg/mL) (red).

**Figure 2:**
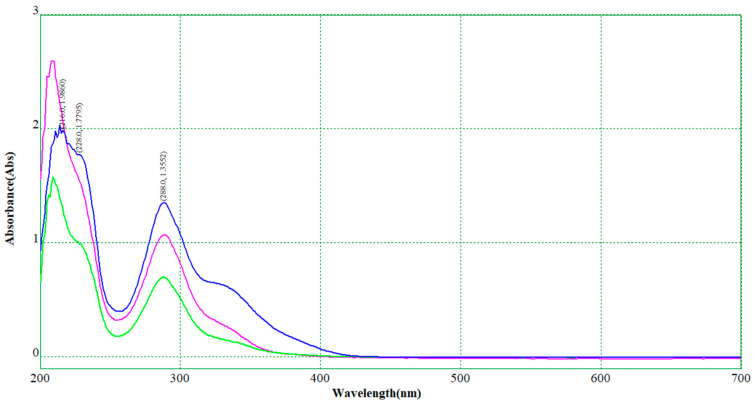
Superimposed UV–Vis spectra for *S. marianum* extract (example for a 33-fold diluted extract, pink), standard silibinin diastereomer mixture, and silymarin mixture solutions (7.5 μg/mL and 9.0 μg/mL, blue and green).

The extracts of *M. chamomilla* (flowers, leaves, roots, and stems) and *S. marianum* (seeds) were obtained at final raw volumes of 27.0–30.5 and 26.5–30.0 mL, respectively (see Table S2 in the Supplementary Material file). The extraction conditions were moderate (<60 °C for 1.5 h) in order not to promote degradation of the antioxidant compounds, namely flavonoids such as chrysin, quercetin, and rutin, or flavonolignans such as silibinins. Moreover, the estimation of the overall antioxidant contents in *M. chamomilla* and *S. marianum* extracts was performed by the spectrophotometric UV–Vis method, a fast and non-destructive technique. These antioxidant contents were expressed as chrysin or rutin in *M. chamomilla* extracts. For the *S. marianum* extracts, the flavonolignan contents were expressed as silibinin (based on either the standard silibinin diastereomer mixture of 98% or the standard silymarin mixture of 64.7%; see Section 3). Determinations were based on specific standard curves obtained at 322 nm for chrysin and rutin (a mixture that better fits the extract absorbance behavior) and 288 nm for silibinin (mean values). The wavelength for flavonoids was selected based on the specific maximum absorbance of *M. chamomilla* extract and the chrysin-rutin mixture at a mass ratio of 8.8:1 (see Equations (1) and (2) and Figure 1). On the other hand, the standard curves for silibinin (based on the silibinin diastereomer mixture and silymarin mixture) are emphasized in Equations (3) and (4), as well as Figure 2. Both chrysin-rutin and silibinin standard systems have similar spectrophotometric behavior with the *M. chamomilla* and *S. marianum* extracts, respectively. The liquid chromatographic technique was not used at this stage due to its main disadvantages regarding such a study, namely its time-consuming nature and possible degradation/hydrolysis during analysis by water/acidic based solvent mixtures.

Conc. (μg/mL) = 85.7(±0.00)·Abs._(@322)_, for rutin (1)

*n* = 6, *r*^2^ = 0.9999, *p* < 0.00001

Conc. (μg/mL) = 29.4(±0.00)·Abs._(@322)_, for chrysin (2)

*n* = 5, *r*^2^ = 1.0000, *p* < 0.00001

Conc. (μg/mL) = 7.136 (±0.040)·Abs._(@288)_, for silibinin (diastereomer mixture) (3)

*n* = 6, *r*^2^ = 0.9996, *p* < 0.00001

Conc. (μg/mL) = 13.066 (±0.038)·Abs._(@288)_, for silymarin mixture (4)

*n* = 9, *r*^2^ = 0.9999, *p* < 0.00001

**Figure 3:**
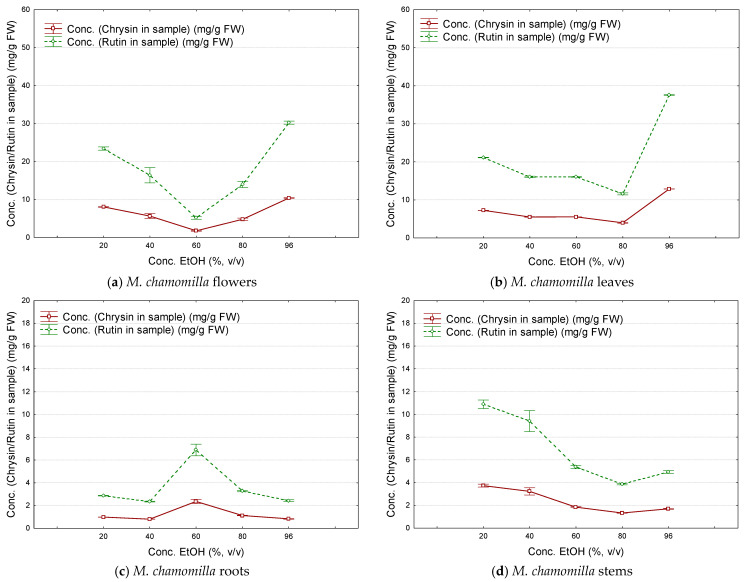
Variation of the estimated flavonoid content in the fresh samples (as chrysin—continuous line, and as rutin—dashed line, mg/g fresh weight) with the ethanol concentration (*v*/*v*) used for obtaining the *M. chamomilla* extracts (number of replicate determinations *n* = 9; error bars were determined from standard errors using a coefficient of ±0.95).

The overall estimation of the flavonoid contents (expressed as chrysin or rutin) revealed that they significantly differed between the flowers/leaves and roots/stems extracts of fresh samples. They were determined using Equations (1) and (2) and various extract dilutions (dilution levels of 0.005 to 0.08). The variation of the estimated flavonoid content (determined as chrysin or rutin in the fresh flowers, leaves, roots, and stems) with the ethanol concentration differs for flowers and leaves in comparison with roots and stems (Figure 3a–d). The maximum flavonoid content for *M. chamomilla* flowers was obtained using ethanol 96% as extraction solvent (8.24 ± 4.69 mg chrysin/g fresh weight, FW; higher values were obtained using rutin as standard: 24.02 ± 13.66 mg/g). On the other hand, roots and stems had lower flavonoid contents, as determined from extracts obtained with ethanol at 20–96% concentrations (1.14–2.75 mg/g FW as chrysin and 4.01–8.01 mg/g as rutin). Results obtained for the estimated flavonoid content (as chrysin or rutin, mg/g FW) of *M. chamomilla* flowers, leaves, roots, and stems using ethanol of various concentrations (*v*/*v*) for extraction are presented in the Supplementary Material file (Table S3). There were only few studies regarding the quantification of polyphenolic contents in specific parts of *M. chamomilla*. They are especially focused on flowers, using liquid chromatographic techniques. Generally, flavonoid contents were determined in dry extracts, such as chamomile ligulate flower extract obtained by subcritical water extraction. Rutin was the main flavonoid glycoside compound identified in extracts at 10 bar pressure (0.23 mg/kg dry extract), while chrysin had the highest concentration in the dry extract obtained at a pressure of 30 bar (1.99 mg/kg). However, higher concentrations of flavonoid aglycones and glycosides were obtained at increased extraction pressure values (1345 mg apigenin/kg at 45 bar, and 1075 mg luteolin 7-*O*-glucoside/kg at 30 bar) [7]. These values were higher if the extraction temperature was increased from 65 to 85 °C. At higher temperatures, the flavonoid aglycones become more concentrated due to partial hydrolysis of the corresponding glycosides during extraction. It is the case of luteolin 7-*O*-glucoside (1101 mg/kg dry extract obtained at 85 °C) and its aglycone, luteolin (97.2 mg/kg at 115 °C and only 55.2 mg/kg at 85 °C) [11]. Apigenin glycosides were also quantified in dry *M. chamomilla* ligulate flowers during flowering. Apigenin 7-*O*-glucoside was determined in the range of 11.38–32.4 mg/g, but the corresponding 6″-malonyl, 6″-caffeoyl, and 4″-acetyl-6″-malonyl-glucosides were also identified [16].

**Figure 4:**
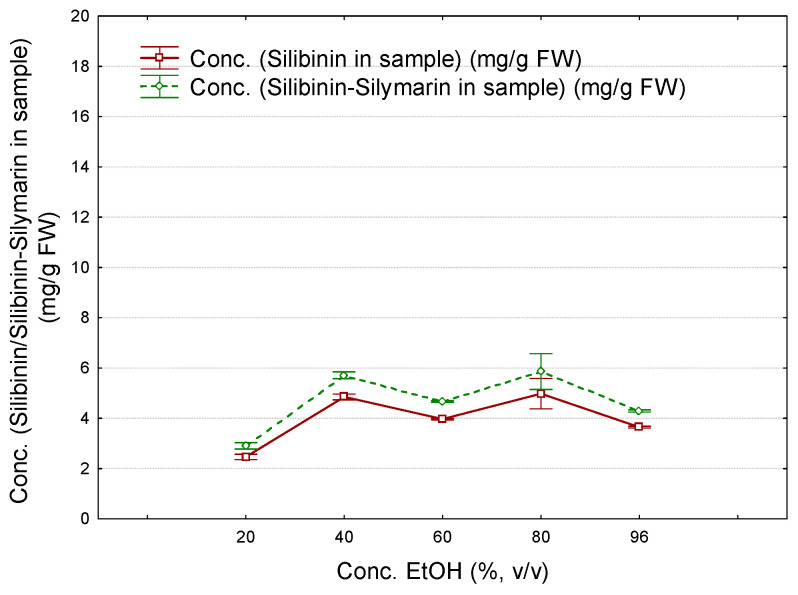
Variation of the estimated silibinin content in the fresh samples (as silibinin based on silibinin standard diastereomer mixture—continuous line, and as silibinin based on standard silymarin mixture—dashed line, mg/g FW) with the ethanol concentration (*v*/*v*) used for obtaining the *S. marianum* extracts (number of replicate determinations *n* = 9; error bars were determined from standard errors using a coefficient of ±0.95).

In a similar manner, the estimated silibinin content of the raw *S. marianum* seeds was determined on the basis of the silibinin diastereomer mixture and silymarin mixture standard curves, using ethanol of various concentrations for extraction (Figure 4). Except for ethanol 20%, all other solvents of 40–96% provide silibinin contents in a very narrow range (mean values of 6.74 ± 0.65 mg/g FW by using silibinin diastereomer standard mixture and 7.93 ± 0.77 mg/g FW by using silymarin standard mixture). These values were approximately equal if ethanol 20% was used for extraction (3.00 ± 0.76 and 3.53 ± 0.90 mg/g FW, respectively). All results obtained for the silibinin content of *S. marianum* samples are presented in Table S4, Supplementary Material file. The results are in good agreement with the other studies related to the silibinin content of *S. marianum* seeds. The silibinin content ranges from 3086 to 9499 mg/kg, depending on genotype [24]. In another study, the silibinin content of the dry extract was in the range of 3.46–10.57 g/100 g, while the total flavonolignans were quantified in the dry extract at 20.47–34.41 mg/100 g [20].

### 2.2. Obtaining of β-Cyclodextrin/M. chamomilla Extract and β-Cyclodextrin/S. marianum Extract Complexes

β-CD/vegetable extract complexes were obtained by the co-crystallization method using equal volumes of β-CD water solution and ethanolic extract. Only extracts obtained with ethanol 96% were used for β-CD complexation. Due to the complexity of antioxidant compounds in the vegetable extracts, the recovering yields of the β-CD/vegetable extract complexes were determined as the dried complex mass divided by the sum of β-CD hydrate mass and antioxidant mass (estimated as rutin for *M. chamomilla* extract and as silibinin for *S. marianum* extract) and expressed as percent. Recovering yields for β-CD/*M. chamomilla* extract complexes were in a narrow range of 55.71–61.87%. On the other hand, these yields were significantly higher for β-CD/*S. marianum* extract complexes (75.72%), which were slightly lower than for the corresponding β-CD/standard silibinin diastereomer mixture and β-CD/silymarin mixture complexes (87.03 and 86.47%, respectively). Quantities, volumes, and recovering yields for all synthesized complexes are presented in Table S4 (Supplementary Material file).

**Figure 5:**
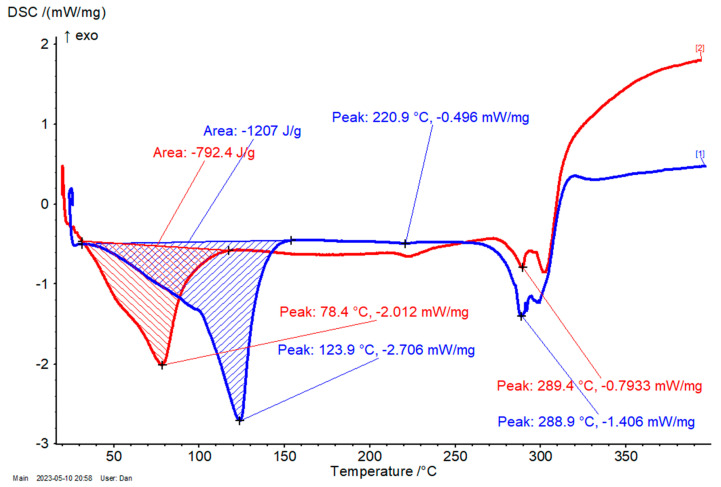
DSC results for the β-CD/*M. chamomilla* flower extract complex (red) and β-CD hydrate (blue); the DSC conditions were set from 20 to 400 °C, with a heating rate of 4 °C/min, under nitrogen (purge and flow).

**Figure 6:**
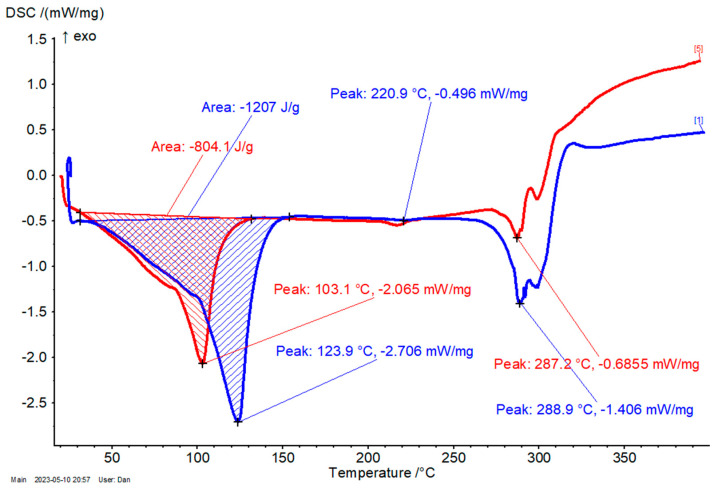
DSC results for the β-CD/*M. chamomilla* stem extract complex (red) and β-CD hydrate (blue); the DSC conditions were set from 20 to 400 °C, with a heating rate of 4 °C/min, under nitrogen (purge and flow).

The thermal stability of complexes was determined by differential scanning calorimetry (DSC). Two main regions were identified: (1) the temperature range of 20–150 °C, where the calorimetric effect appears, which corresponds to the dissociation of water and ethanol molecules from the β-CD complex; and (2) the temperature range of ~270–320 °C, where the decomposition of β-CD and encapsulated antioxidant compounds takes place. The β-CD/*M. chamomilla* flower extract complex has a significantly lower calorimetric effect for the first region in comparison with the β-CD hydrate (792 and 1207 J/g, respectively; Figure 5). Moreover, the dissociation of the crystallization water/ethanol has the highest rate at 78.4 °C for the complex, while this value is much higher for β-CD hydrate (123.9 °C). This is especially due to the partial replacement of strongly retained water molecules into the β-CD complex in comparison with the β-CD hydrate. No significant differences were observed for the temperature corresponding to the maximum rate of decomposition (~289 °C for both cases). For the other complexes, the temperature for the first region is lower than the corresponding β-CD hydrate, but the differences are smaller, especially for β-CD complexes with leaf and root extracts. In these cases, the calorimetric effect is also closer to the β-CD hydrate. They were in the range of 119.8–123.4 °C and 1031–1155 J/g, respectively. The β-CD/*M. chamomilla* stem extract complexes have intermediate values for these parameters (103.1–85.9 °C and 804.1 J/g, respectively; see Figure 6). The temperature decomposition of these complexes was in the same narrow range of 278.7–285.2 °C. Another calorimetric peak appears at approximately 221 °C for β-CD hydrate, which is slightly modified after complexation. It is due to the transition of the anhydrous β-CD from the crystalline to the amorphous forms (see also Figures S1 and S2 in the Supplementary Material file).

**Figure 7:**
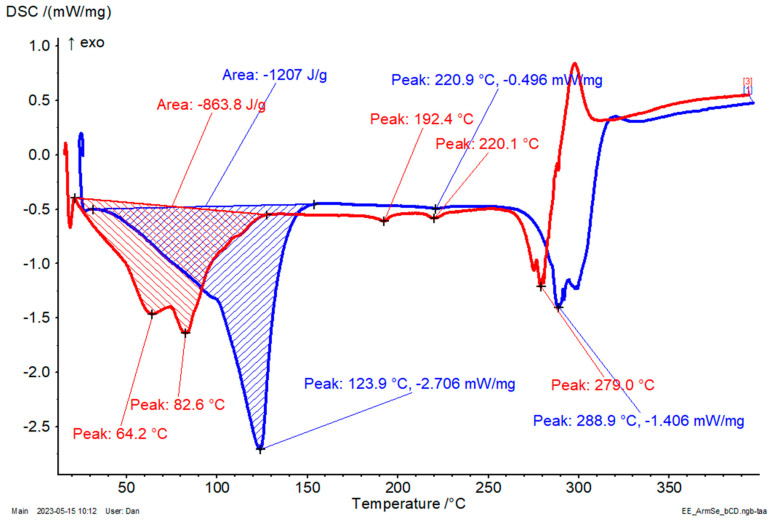
DSC results for the β-CD/*S. marianum* seed extract complex (red) and β-CD hydrate (blue); the DSC conditions were set from 20 to 400 °C, with a heating rate of 4 °C/min, under nitrogen (purge and flow).

Similar behavior was observed for β-CD/*S. marianum* seed extract complex, but with a significantly lower value for the temperature corresponding to water/ethanol molecule release. Moreover, there are two peak temperatures at 64.2 and 82.6 °C, which are due to the release of surface water and strongly retained water molecules [47,48]. The endothermal calorimetric effect is close to that corresponding to β-CD/*M. chamomilla* flower and stem extract complexes (863.8 J/g, in comparison with 792.4–804.1 J/g). The maximum decomposition rate appears at a slightly lower value of 279 °C, while the transition effects were observed at 192.4 and 220.1 °C (Figure 7).

This calorimetric behavior was also observed in other studies for CD/antioxidant complexes based on *Capsicum annuum* ethanolic extract, flavonoids such as chrysin, naringenin, hesperetin, apigenin, luteolin, and rutin, as well as flavonolignans, namely silibinin [32,49,50,51]. Moreover, CD/hydrophobic compound complexes including standard fatty acids or hydrophobic mixtures (essential oils, vegetable oils/lipid fractions, or fish oils) reveal a decrease in the calorimetric effect corresponding to water release as well as a change in the endo-exothermic calorimetric effect of the crystalline-amorphous transition processes [52,53,54,55,56,57,58,59].

The water content of β-CD complexes can be selectively determined using the volumetric Karl Fischer titration (KFT) [60]. Thus, the ethanol molecules that can remain in the complex during preparation will not be accounted by using this method. Water content values were in the range of 10.56–12.37% for complexes, significantly lower than β-CD hydrate (15.27%, Table 1). β-CD complexes with silibinin diastereomer standard mixture or silymarin standard mixture had KFT water content values close to the upper limit of the above-mentioned range (12.50 and 12.71%, respectively, Table 1). The KFT water content well correlates with the DSC endothermic calorimetric peak corresponding to the dissociation of water/ethanol molecules from the β-CD complexes, with a good determination coefficient of *r*^2^ = 0.690 (see Equation (5)). Both KFT water content/titration behavior and KFT–DSC correlations were in accordance with other studies related to CD/flavonoid, CD/vegetable extract, CD/fish oil, or CD/essential oil complexes [32,49,55,56,59].

*Water content* (by KFT, %) = 4.529(±2.567) + 0.0077(±0.0026)·*DSC_Area (20–150 °C)_* (5)

*n* = 6, *r*^2^ = 0.690, *p* < 0.05, *s* = 1.05

### 2.3. Controlled Release of Antioxidants from β-Cyclodextrin Complexes and Transdermal Pharmaceutical Formulations Containing β-Cyclodextrin Complexes

β-CD forms stable molecular inclusion complexes with a wide range of compounds having various structural characteristics. Flavonoid aglycones such as quercetin, naringenin, hesperetin, kaempferide, and chrysin are complexed by β-CD with stability constants of 602/3345, 235, 355, 1541, and 574 M^−1^ [36,61,62,63]. On the other hand, the more hydrophilic flavonoid glycoside rutin has a stability constant for the β-CD/rutin inclusion complex of 260–265 M^−1^ [39,61,64]. The last stability constant is lower due to the reduced level of hydrophobic interaction of flavonoid glycoside with the inner cavity of the β-CD in comparison with the case of less hydrophilic flavonoid aglycones. Similar behavior was observed for β-CD/silymarin (silibinin) molecular inclusion complexes, which had an apparent stability constant of 722 M^−1^ [44]. Highly hydrophobic molecules such as fatty acids, lipid sterols, and terpenes provide much higher β-CD binding constants. Thus, β-CD/lauric acid and β-CD/cholesterol complexes had a 1:1 binding constant of 28,000 and 23,190 M^−1^, respectively [65,66]. On the other hand, monoterpenes such as β-pinene, myrcene, and limonene have β-CD formation constant values of 4646, 1267, and 2605 M^−1^, while the sesquiterpene β-caryophyllene has a significantly higher value of 23,032 M^−1^ [67]. Consequently, more hydrophilic flavonoid aglycones and especially glycosides, as well as flavonolignans, will be easier released from the β-CD complex in proper solvents or matrices. In this study, the controlled release of flavonoids (expressed as rutin or chrysin) from β-CD/*M. chamomilla* extract complexes and flavonolignans (expressed as silibinin) from β-CD/*S. marianum* extract complexes was estimated by spectrophotometric analysis. The solvents used for the controlled release studies were ethanol at concentrations of 20, 60, and 96% for the crystalline β-CD/vegetable extract complexes, as well as saline solution (0.9% NaCl at pharmaceutical grade) and the above-mentioned ethanol solutions for the studies on transdermal pharmaceutical formulations.

**Figure 8:**
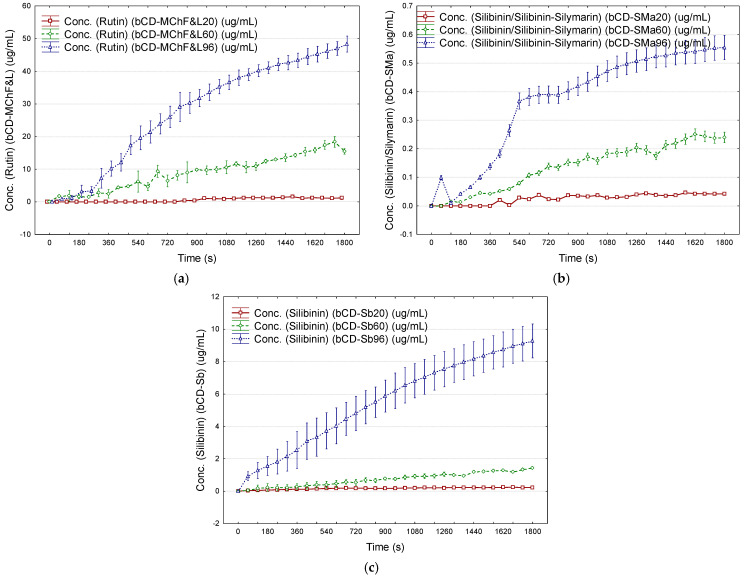
Controlled release of the estimated antioxidant compounds from the β-cyclodextrin/*M. chamomilla* flower and leaf extract complexes (expressed as rutin, μg/mL) (**a**), from the β-cyclodextrin/*S. marianum* seed extract complexes (expressed as silibinin, μg/mL) (**b**), and from β-cyclodextrin/silibinin or silymarin complexes (expressed as silibinin, μg/mL) (**c**) in ethanol 20% (brown), ethanol 60% (green), and ethanol 96% (bleu); number of replicate determinations *n* = 2; error bars were determined from standard errors using a coefficient of ±0.95. Codes for complexes are: *bCD_MChXY*—β-cyclodextrin/*M. chamomilla* L. extract; *bCD_SMaY*—β-cyclodextrin/*S. marianum* L. extract; *X* stands for the plant part, *X* = *F*—flowers, *L*—leaves, *R*—roots, or *S*—stems; *bCD_Sb/SmY*—β-cyclodextrin/silibinin or β-cyclodextrin/silymarin complexes; *Y* stands for the concentration of ethanol solution used for controlled release (20, 60, and 96%, *v*/*v*).

The estimated flavonoids were released in higher amounts, especially in ethanol 96%, in comparison with ethanol 20 and 60%. The maximum cumulative content of flavonoids (expressed as μg rutin/mL after 15 min) obtained for the controlled release from β-CD/*M. chamomilla* flower and leaf extract complexes was 32.5 μg/mL (mean value) in ethanol 96%. On the contrary, these values were 10.4 and 0.8 μg/mL if ethanol 60 and 20% were used. By comparison, the maximum flavonoid content of only 18.0 μg/mL was obtained by controlled release from β-CD/*M. chamomilla* root and stem extract complexes (Figure 8a and Figures S3, S4, S36–S38 in the Supplementary Material file). Similar behavior was observed for the controlled release of silibinin from the β-CD/*S. marianum* extract complexes. The estimated maximum silibinin content was obtained by controlled release in ethanol 96% (0.56 μg/mL), compared with ethanol 20 and 60% (<0.24 μg/mL). By comparison, standard silibinin was easily released from the β-CD/silibinin complexes (mean value of 9.3 μg/mL in ethanol 96% after 15 min) (Figure 8b,c and Figures S5–S7).

**Figure 9:**
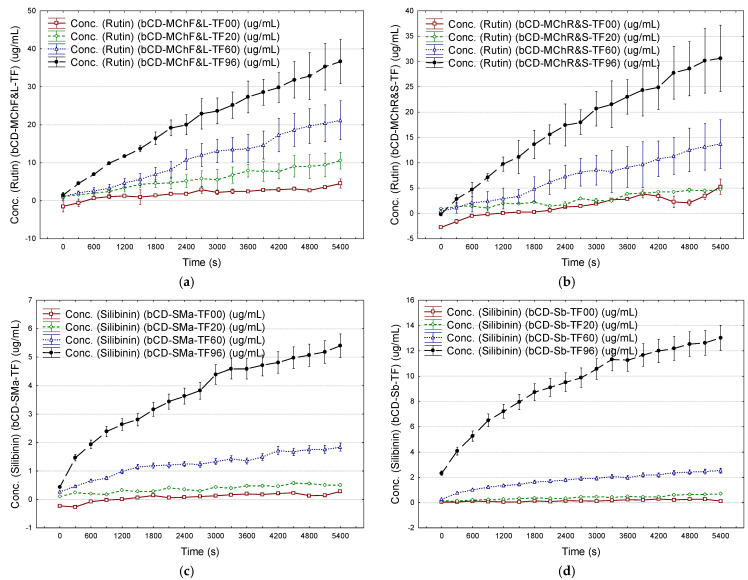
Controlled release of the estimated antioxidant compounds from the transdermal pharmaceutical formulations containing β-cyclodextrin/*M. chamomilla* flower and leaf extract complexes (expressed as rutin, μg/mL) (**a**), from the transdermal pharmaceutical formulations containing β-cyclodextrin/*M. chamomilla* root and stem extract complexes (expressed as rutin, μg/mL) (**b**) from the transdermal pharmaceutical formulations containing β-cyclodextrin/*S. marianum* seed extract complexes (expressed as silibinin, μg/mL) (**c**), and from the transdermal pharmaceutical formulations containing β-cyclodextrin/silibinin complexes (expressed as silibinin, μg/mL) (**d**), in saline solution (brown), ethanol 20% (green), ethanol 60% (bleu), and ethanol 96% (black); number of replicate determinations *n* = 2; error bars were determined from standard errors using a coefficient of ±0.95. Codes for the transdermal pharmaceutical formulations based on cyclodextrin complexes are: *bCD_MChX_TFY*—transdermal pharmaceutical formulation based on β-cyclodextrin/*M. chamomilla* L. extract; *bCD_SMa_TFY*—transdermal pharmaceutical formulation based on β-cyclodextrin/*S. marianum* L. extract; *X* stands for the plant part; *X* = *F*—flowers, *L*—leaves, *R*—roots, or *S*—stems; *bCD_Sb/Sm_TFY*—transdermal pharmaceutical formulation based on β-cyclodextrin/silibinin or β-cyclodextrin/silymarin complexes; *Y* stands for saline solution (“00”) or the concentration of ethanol solution used for controlled release (20, 60, and 96%, *v*/*v*).

**Figure 10:**
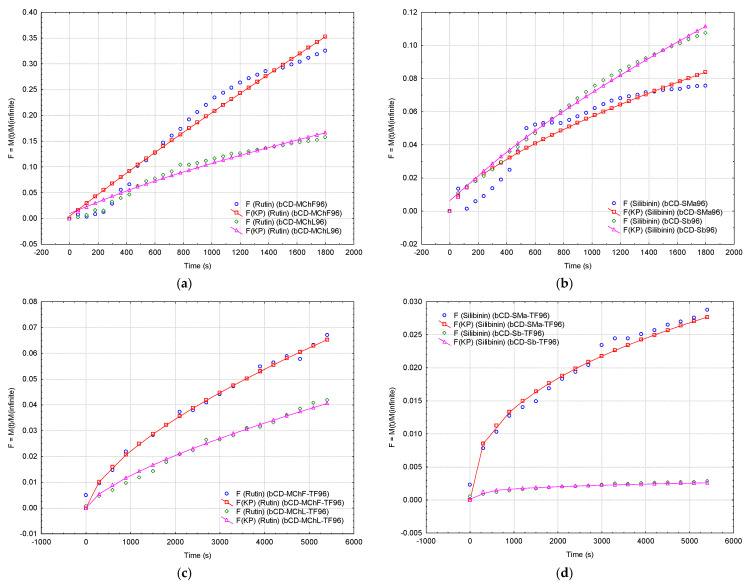
Korsmeyer–Peppas modeling (*M_t_*/*M_∞_* = *k(_KP)_*·*t^n^*) of the controlled release of the estimated antioxidants from β-cyclodextrin complexes and from transdermal pharmaceutical formulations based on β-cyclodextrin complexes. *M_t_* and *M_∞_* stand for the amount (mg) of antioxidant released from the complex at the time *t* (s) and the total amount, respectively. The Korsmeyer–Peppas kinetic constant, *k*_(*KP*)_, and the diffusional exponent, *n*, were determined by fitting the experimental data (least squares approximation; continuous lines). Codes for complexes are: *bCD_MChXY*—β-cyclodextrin/*M. chamomilla* L. extract; *bCD_SMaY*—β-cyclodextrin/*S. marianum* L. extract; codes for the transdermal pharmaceutical formulations based on cyclodextrin complexes are: *bCD_MChX_TFY*—transdermal pharmaceutical formulation based on β-cyclodextrin/*M. chamomilla* L. extract; *bCD_SMa_TFY*—transdermal pharmaceutical formulation based on β-cyclodextrin/*S. marianum* L. extract; *X* stands for the plant part, *X* = *F*—flowers, *L*—leaves, *R*—roots or *S*—stems; *bCD_Sb/Sm_TFY*—transdermal pharmaceutical formulation based on β-cyclodextrin/silibinin or β-cyclodextrin/silymarin complexes; *Y* stands for saline solution (“00”) or the concentration of ethanol solution used for controlled release (20, 60, and 96%, *v*/*v*). Experimental plots and Korsmeyer–Peppas model fitting (plots and continuous line) for the controlled release of the estimated antioxidants from *bCD_MChF* and *bCD_MChL* complexes in ethanol 96% (**a**), from *bCD_SMa* and *bCD_Sb* complexes in ethanol 96% (**b**), from transdermal pharmaceutical formulations based on *bCD_MChF* and *bCD_MChL* complexes in ethanol 96% (**c**), and from transdermal pharmaceutical formulations *bCD_SMa* and *bCD_Sb* complexes in ethanol 96% (**d**).

The mathematical modeling of the controlled release of antioxidants from β-CD complexes and transdermal pharmaceutical formulations can provide information about the releasing mechanism (Figure 8a–c, Figure 9a–d and Figure 10a–d). The Korsmeyer–Peppas model has been considered [68]. Values of the diffusional exponent *n* of 0.5 and 1 reveal Fickian diffusion and Case II transport (relaxation release), respectively. A qvasi-Fickian diffusion (partial diffusion) appears if *n* < 0.5, while the non-Fickian (anomalous) diffusion appears for *n* ∈ (0.5, 1), which means that compounds are released by both diffusion and relaxation (erosion). Finally, the Super Case II transport is revealed by a diffusional exponent of *n* > 1 [68,69].

The best results were obtained for ethanol 60 and 96% (best fitting of the experimental data; see Section 3.5). Thus, Case II transport (*n* ≅ 0.92) and super-Case II transport (*n* > 1) mechanisms were observed for controlled release of estimated antioxidants from β-CD/*M. chamomilla* flower extract complexes in ethanol 96% and ethanol 60%, respectively (*r*^2^ of 0.985 and 0.966). On the contrary, a non-Fickian diffusion (*n* ≅ 0.76) was observed for the cases of β-CD/*M. chamomilla* leaf extract complexes studied in ethanol 60 and 96% (*r*^2^ of 0.97–0.99). The release mechanisms of the estimated antioxidants from β-CD/*M. chamomilla* root and stem extract complexes were Case II transport for releasing in ethanol 60 and 96% (root extract case, *n* = 1.02–1.04, *r*^2^ ≅ 0.98), or in ethanol 60% for the case of stem extract-based complexes (*n* = 0.94, *r*^2^ = 0.97). The silibinin release mechanism from β-CD/*S. marianum* extract complexes was non-Fickian for all ethanol concentrations (*n* = 0.66–0.81 and *r*^2^ = 0.90–0.98), such as in the case of β-CD/silibinin complexes in ethanol 96% (*n* = 0.76, *r*^2^ = 0.998). A comparison between the Korsmeyer–Peppas model and experimental data is emphasized in Figure 10a,b for the controlled release of estimated antioxidants from β-CD/*M. chamomilla* flower and leaf extract complexes, β-CD/*S. marianum* extract, and β-CD/silibinin complexes in ethanol 96%. All values for the kinetic constant, *k*_(*KP*)_, and diffusional exponent, *n*, as well as the values for the coefficient of determination, *r*^2^, corresponding to the fitting of the Korsmeyer–Peppas model with the experimental data, are presented in Table S6 (Supplementary Material file).

The same Korsmeyer–Peppas model was used for the evaluation of the release mechanism of antioxidants from transdermal pharmaceutical formulations. In all cases, the diffusional exponent was *n* < 1, except in the case of β-CD/*M. chamomilla* flower extract complexes studied in ethanol 60%, where *n* = 1 (Case II transport mechanism). However, the diffusional exponent had values of 0.5–1 for all other β-CD/*M. chamomilla* extract complex-based formulations studied in ethanol 60 and 96%, revealing a non-Fickian diffusion for the antioxidant release (Figure 9a,b and Figure 10c, as well as Table S7 and Figures S8–S23, S39 and S40 in the Supplementary Material file). On the contrary, a qvasi-Fickian diffusion of the estimated antioxidants (silibinin) in ethanol of 20–96% concentrations from all transdermal pharmaceutical formulations based on β-CD/*S. marianum* extract, β-CD/silibinin, and β-CD/silymarin complexes was observed. The highest *n* values and the best correlations were obtained if ethanol 96% was used (*n* = 0.14–0.40, *r*^2^ = 0.88–0.99, Figure 9c,d and Figure 10d). All results are presented in Table S7, Figures S24–S27 for the case of β-CD/*S. marianum* extract complexes, and Figures S28–S35 and S41 for the case of β-CD/silibinin or silymarin complexes (Supplementary Material file).

## 3. Materials and Methods

### 3.1. Plant Samples and Chemicals

Plant materials (*M. chamomilla* L. and *S. marianum* L.) were collected from specific locations in the west of Romania. Thus, *M. chamomilla* L. plants were harvested during autumn seasons of the last years from the Salonta wild region (46°48′0″ N, 21°39′00″ E, Bihor County, Romania). Flowers, leaves, roots, and stems were carefully separated and stored as fresh samples at −20°C until extractions. Only the seeds of *S. marianum* L. collected in the same period from the Macea region (46°23′12″ N, 21°18′39″ E, Arad County, Romania) were used. The main antioxidant compounds in extracts were chrysin, quercetin, and rutin hydrate. They were used as standard compounds from the flavonoid class and had purities of 97%, 98%, and 94%, respectively (Sigma-Aldrich, St. Louis, MO, USA). On the other hand, standard compounds from the flavonolignan class, namely silibinin and silymarin, had contents of 98% and 64.7% silibinins, respectively (silymarin composition was based on spectrophotometric analysis in this study; Sigma-Aldrich, St. Louis, MO, USA). β-Cyclodextrin hydrate was purchased from CycloLab (Budapest, Hungary) and had >98% purity and a water content of 12.4% (loss on drying, according to the manufacturer). Ethanol 96% (*v*/*v*) used for CD complexation and controlled release studies was obtained from ChemiCal^®^ Co. (Iaşi, Romania).

### 3.2. Obtaining M. chamomilla and S. marianum Extracts

Fresh samples of *M. chamomilla* (flowers, leaves, roots, and stems) and *S. marianum* (seeds) were well grounded in a mortar. Approximately 5 g of every sample was immediately weighted into a 150 mL extraction flask. The samples were extracted with 70 mL of ethanol at various concentrations (20, 40, 60, 80, and 96%, *v*/*v*, a solid:solvent ratio of ~1:14) for 1.5 h by continuous stirring on a water bath at 55–60 °C [14]. The extracts were subjected to spectrophotometric analysis after filtration (see below) or were subjected to concentration at a third of the volume for CD nanoencapsulation. All data and the raw results of the extraction of extracts are presented in the Supplementary Material file (Table S1).

### 3.3. Obtaining of β-cyclodextrin/M. chamomilla Extract and β-cyclodextrin/S. marianum Extract Complexes

β-CD/vegetable extract complexes were obtained using the co-crystallization method. Approximately 0.5 mmoles of β-CD hydrate were dissolved in 4 mL water in a 20 mL crystallization flask, which was equipped with cooling-heating mantle, reflux condenser, and magnetic stirring system. The solution was heated to 50 °C, and then 4 mL of vegetable extract was added dropwise through the top of the condenser for 15 min. The solution became turbid during the controlled cooling to room temperature (cooling rate of ~7.5 °C/h) and the suspension of the complex was stored at 4 °C overnight. The crystallized complex was filtered through Whatman^®^ filter paper No 1 (Merck KGaA, Darmstadt, Germany) and washed with 1 mL ethanol 96%. After drying at room temperature, the complex crystals were weighted, sealed, and stored at 4 °C until further analysis.

### 3.4. Obtaining Transdermal Pharmaceutical Formulations

The controlled release behavior of β-CD/vegetable extract complexes was evaluated from the standard transdermal pharmaceutical formulations (transdermal cream consisting of 10% lanolin and 90% vaseline, Herbavit, Romania). The β-CD complex was incorporated into the transdermal cream samples by mixing for 1 h at a complex:cream ratio of 1:20. All determinations were performed against a blank sample (without complexes).

### 3.5. Spectrophotometric (UV–Vis) Analysis and Controlled Release Measurements

Spectrophotometric analysis was performed for extracts and standard compound solutions at various concentrations, as well as for the evaluation of their release from β-CD/extract complexes and pharmaceutical formulations. This is a fast and non-destructive method for the evaluation/estimation of the overall release of biologically active compounds from nanoparticles and pharmaceutical formulations. A CamSpec 501 equipment (CamSpec Ltd., Cambridge, UK) was used. The absorbance values of the samples were monitored at specific wavelengths (322 and 288 nm for *M. chamomilla* and *S. marianum* extracts, respectively) using the Wavelength Scan Measurement and/or Time Scan Measurement modules. The monitoring time was set at 30 min for controlled release from the crystalline β-CD/vegetable extract complexes in ethanol solutions of 20, 60, and 96% concentrations (*v*/*v*). The controlled release of the estimated bioactive compounds from the transdermal pharmaceutical formulations based on β-CD/vegetable extract complexes was studied for 90 min at the same wavelengths, using even ethanol (20, 60, and 96%) or saline solution (0.9% NaCl, pharmaceutical grade) as solvent media. The acquisition and handling of the UV–Vis data were performed with the UV–Vis Analyst version 4.67 software from the same manufacturer. In all cases, the appropriate solvent mixture was used as the blank sample.

The controlled release of the estimated antioxidants from β-CD complexes or from transdermal pharmaceutical formulations based on β-CD complexes was modeled using the Korsmeyer–Peppas model (Equation 6) [68]:

*F* = *M_t_/M_∞_* = *k*_(*KP*)_·*t^n^* (6)

The model uses the ratio (*F*) between the antioxidant (biologically active compound or drug molecule) amount that is released at the moment *t* (s) and the total amount of antioxidant in the sample (*M_t_/M_∞_*). This ratio is correlated with kinetic constant *k*_(*KP*)_ (expressed as s^−n^) and the time at the power of a diffusional exponent, *t^n^*. Parameters *k*_(*KP*)_ and *n* reveal the controlled release mechanism and were determined from the experimental data by fitting (least squares approximation). All values for these parameters, as well as those for the coefficient of determination, *r*^2^, can be found in the Supporting Material file (Tables S6 and S7).

### 3.6. Differential Scanning Calorimetry (DSC)

DSC analysis was performed for all solid materials (β-CD/vegetable extract complexes and β-CD hydrate) in order to evaluate the calorimetric effects during heating. A DSC Netzsch 204 F1 Phoenix equipment (Netzsch Group, Selb, Germany) was used, with a temperature range of 20–400 °C and a heating rate of 4 °C/min. Nitrogen protection and purge flow were set at 20 mL/min. The sample masses were 4.3–8.0 mg for complexes and 9.8–10.0 mg for β-CD. DSC analysis was performed in open Al crucibles using a reference crucible of 39.4 mg. The Netzsch Proteus-Thermal Analysis ver. 6.1 software (Netzsch Group, Selb, Germany) was used for acquisition and handling of the DSC data.

### 3.7. Karl Fischer Water Titration (KFT)

The water content of complexes and β-CD hydrate was determined using the volumetric KFT method. A KF Titrando 701 apparatus having a Metrohm 10 dosing unit and coupled with a Ti Stand 703 mixing unit (Metrohm AG, Herisau, Switzerland) was used. The bi-component KFT technique was applied, with the component 1—Titrant 5 apura^®^ (Merck KGaA, Darmstadt, Germany) having a titer of 3.6357 ± 0.0195 mg/g at the analysis period. The titer was determined using the water standard 1% apura^®^ (Merck KGaA, Darmstadt, Germany). Component 2—Solvent apura^®^—was used as a working medium and was purchased from the same manufacturer (Merck KGaA, Darmstadt, Germany). Sample masses were in the range of 20.0–40.0 mg. The following KFT parameters were used: *I*(pol) 50µA, end point 250 mV, maximum titration rate 5 mL/min, stop criteria—drift with a value of 15 µL/min. The extraction time was approximately 300 s. All determinations were performed in triplicate.

### 3.8. Statistical Analysis

All values obtained for multiplicate determinations (analysis and/or synthesis) were provided as mean ± standard deviation (SD). The Basic Statistics&Tables and One-way ANOVA modules in Statistica 7.1 (StatSoft, Inc., Tulsa, OK, USA) software were used. The regression analysis was performed with the Multiple Linear Regression module from the same package. Pearson correlational coefficient, *r*, the coefficient of determination, *r*^2^, *p*-values, *F*-test for the regression equation, and standard errors for both equations and coefficients have been considered.

## 4. Conclusions

A series of β-CD/chamomile (*Matricaria chamomilla* L.) and β-CD/milk thistle (*Silybum marianum* L.) extract complexes were synthesized and evaluated for their controlled release properties using a fast, cheap, and non-destructive method, namely spectrophotometric analysis. This is the first study on the controlled release of the estimated antioxidants from such complexes and pharmaceutical formulations containing the above-mentioned complexes. Extracts were obtained with ethanol solutions at various concentrations and moderate temperatures in order to reduce the level of degradation of labile antioxidant compounds. High estimates of antioxidant compounds (such as rutin or chrysin for *M. chamomilla* flowers and leaves, as well as silibinin for *S. marianum*) in extracts or raw vegetable samples were obtained if ethanol at concentrations of 80–96% was involved. These extracts were used for obtaining β-CD complexes using the co-crystallization method, which provides high recovering yields, especially for the β-CD/*S. marianum* extract complex. The thermal stability of all complexes is similar to that of β-CD hydrate, according to DSC analysis. However, the hydration water content (especially “strongly bonded”, based on both DSC and KFT analyses) is much lower in complexes, which reveals the formation of molecular inclusion complexes.

The modeling of the controlled release of the estimated antioxidant compounds from β-CD complexes and transdermal pharmaceutical formulations based on these β-CD complexes was an important and innovative part of this study. Different ethanol solutions or saline solutions (releasing solvents with various hydrophobicities) were used for controlled release studies. On the other hand, both β-CD complexes (more hydrophilic) and transdermal pharmaceutical formulations based on β-CD complexes (more hydrophobic) were considered for controlled release studies. First, the release of the estimated antioxidants from β-CD complexes was performed in ethanol of 20, 60, and 96% (*v*/*v*), which have log*P* values of −1.14, −0.74, and −0.37 (estimated from the corresponding mole fractions by using the logarithmic interpolation based on log*P* values of pure components) [70]. The selection of these ethanol concentrations was based on the highly hydrophilic character of β-CD and its complexes (the solubility of β-CD is much higher in water than in ethanol-water mixtures). In the Korsmeyer–Peppas model, β-CD/*M. chamomilla* flower extract complexes reveal Case II transport mechanisms, while the corresponding complexes with leaf extracts indicate non-Fickian diffusion for the controlled release of the estimated antioxidants in ethanol 60 and 96%. The same non-Fickian diffusion was revealed by β-CD/*S. marianum* extract and β-CD/silibinin complexes. On the contrary, the controlled release studies for the model transdermal pharmaceutical formulations based on β-CD/vegetable extract complexes that were performed in the same ethanol (20% to 96%) as well as in saline solution (0.9% NaCl) revealed completely different releasing mechanisms. Almost all formulations based on β-CD/*M. chamomilla* extract complexes and all those based on β-CD/*S. marianum* extract complexes revealed non-Fickian diffusion for the estimated antioxidant release (expressed as rutin and silibinin, respectively). This is most likely due to the completely different hydrophilic/hydrophobic characteristics of matrices. Thus, β-CD complexes have more hydrophilic cyclic oligosaccharides and crystallization water as the main matrix components. Flavonoids and flavonolignans are mainly hydrophilic. As a consequence, *H*-bonding is especially involved in the diffusion of antioxidants into this type of matrix. For transdermal pharmaceutical formulations, fatty acid glycerides and sterol esters with long-chain fatty acids are the main components. Consequently, a competition between the hydrophobic interactions of the hydrophobic components of the matrix and the complexed antioxidants with the hydrophobic inner cavity of β-CD appears. In this case, the controlled release of antioxidants is mainly due to hydrophobic interactions. These differences have been proven in this study by the modeling of the diffusion of the estimated antioxidants into two types of matrices with different hydrophobicities. Results can be further used for studying the particular antioxidant transdermal transport and biological effects of innovatively designed pharmaceutical formulations that can be obtained using “green” methods and materials.

## Figures and Tables

**Table 1:** Water content of β-CD/vegetable extract complexes and β-CD hydrate, obtained by volumetric Karl Fischer titration technique (values are expressed as mean ± standard deviation, SD, *n* = 3).

Code	Description	Water Content (%)
*bCD*	β-Cyclodextrin hydrate	15.27 ± 0.15
*bCD_MChF*	β-Cyclodextrin/*M. chamomilla* flower extract complex	11.05 ± 0.04
*bCD_MChL*	β-Cyclodextrin/*M. chamomilla* leaf extract complex	12.37 ± 0.68
*bCD_MChR*	β-Cyclodextrin/*M. chamomilla* root extract complex	11.90 ± 0.12
*bCD_MChS*	β-Cyclodextrin/*M. chamomilla* stem extract complex	11.31 ± 0.14
*bCD_SMa*	β-Cyclodextrin/*S. marianum* seed extract complex	10.56 ± 0.31
*bCD_Sb*	β-Cyclodextrin/silibinin complex	12.50 ± 0.28
*bCD_Sm*	β-Cyclodextrin/silymarin complex	12.71 ± 0.33

## Data Availability

Not applicable.

## Supplementary Materials

The following are available online at https://www.mdpi.com/article/10.3390/plants12122352/s1. Table S1: Raw data and results for the obtaining of *M. matricaria* extracts. Table S2: Raw data and results for the obtaining of *S. marianum* extracts. Table S3: Results obtained for the flavonoid content (as chrysin or rutin, mg/g FW) of *M. chamomilla* flowers, leaves, roots, and stems using ethanol of various concentrations (*v*/*v*) for extraction. Table S4: Results obtained for the silibinin content (based on both silibinin diastereomer mixture and silymarin mixture, mg/g FW) of *S. marianum* using ethanol of various concentrations (*v*/*v*) for extraction. Table S5: Results obtained for β-cyclodextrin/vegetable extract or β-cyclodextrin/flavonolignan complexes (codes for complexes: *bCD_MChX*—β-cyclodextrin/*M. chamomilla* L. extract; *bCD_SMaX*—β-cyclodextrin/*S. marianum* L. extract; *X* stands for the plant part, *X* = *F*—flowers, *L*—leaves, *R*—roots, or *S*—stems; *bCD_Sb/Sm*—β-cyclodextrin/silibinin or β-cyclodextrin/silymarin complexes). Figure S1: DSC results for the β-CD/*M. chamomilla* leaf extract complex (red) and β-CD hydrate (blue); the DSC conditions were set from 20 to 400 °C, with a heating rate of 4 °C/min under nitrogen (purge and flow). Figure S2: DSC results for the β-CD/*M. chamomilla* root extract complex (red) and β-CD hydrate (blue); the DSC conditions were set from 20 to 400 °C, with a heating rate of 4 °C/min under nitrogen (purge and flow). Figure S3: Superimposed Absorbance versus Time (s) plots for the controlled release studies on β-CD/*M. chamomilla* flower and leaf extracts in ethanol 20% (blue), ethanol 60% (green), and ethanol 96% (turquoise); raw data obtained by monitoring the absorbance of the supernatant solution at 322 nm for 30 min. Figure S4: Superimposed Absorbance versus Time (s) plots for the controlled release studies on β-CD/*M. chamomilla* root and stem extracts in ethanol 20% (blue), ethanol 60% (green), and ethanol 96% (turquoise); raw data obtained by monitoring the absorbance of the supernatant solution at 322 nm for 30 min. Figure S5: Superimposed Absorbance versus Time (s) plots for the controlled release studies on β-CD/*S. marianum* seed extracts in ethanol 20% (blue), ethanol 60% (green), and ethanol 96% (turquoise); raw data obtained by monitoring the absorbance of the supernatant solution at 288 nm for 30 min. Figure S6: Superimposed Absorbance versus Time (s) plots for the controlled release studies on β-CD/silibinin diastereomer mixture complex in ethanol 20% (blue), ethanol 60% (green), and ethanol 96% (turquoise); raw data obtained by monitoring the absorbance of the supernatant solution at 288 nm for 30 min. Figure S7: Superimposed Absorbance versus Time (s) plots for the controlled release studies on β-CD/silymarin standard mixture complex in ethanol 20% (blue), ethanol 60% (green), and ethanol 96% (turquoise); raw data obtained by monitoring the absorbance of the supernatant solution at 288 nm for 30 min. Figure S8: Superimposed UV–Vis spectra for the controlled release studies on the transdermal pharmaceutical formulation based on β-CD/*M. chamomilla* flower extract complex in saline solution; raw data obtained by monitoring the absorbance of the supernatant solution in the range of 250–400 nm for 90 min (a number of 19 spectra were recorded every 5 min, starting from 0 min—the lowest spectra—to 90 min—the highest spectra). Figure S9: Superimposed UV–Vis spectra for the controlled release studies on the transdermal pharmaceutical formulation based on β-CD/*M. chamomilla* flower extract complex in ethanol 20% solution; raw data obtained by monitoring the absorbance of the supernatant solution in the range of 250–400 nm for 90 min (a number of 19 spectra were recorded every 5 min, starting from 0 min—the lowest spectra—to 90 min—the highest spectra). Figure S10: Superimposed UV–Vis spectra for the controlled release studies on the transdermal pharmaceutical formulation based on β-CD/*M. chamomilla* flower extract complex in ethanol 60% solution; raw data obtained by monitoring the absorbance of the supernatant solution in the range of 250–400 nm for 90 min (a number of 19 spectra were recorded every 5 min, starting from 0 min—the lowest spectra—to 90 min—the highest spectra). Figure S11: Superimposed UV–Vis spectra for the controlled release studies on the transdermal pharmaceutical formulation based on β-CD/*M. chamomilla* flower extract complex in ethanol 96% solution; raw data obtained by monitoring the absorbance of the supernatant solution in the range of 250–400 nm for 90 min (a number of 19 spectra were recorded every 5 min, starting from 0 min—the lowest spectra—to 90 min—the highest spectra). Figure S12: Superimposed UV–Vis spectra for the controlled release studies on the transdermal pharmaceutical formulation based on β-CD/*M. chamomilla* leaf extract complex in saline solution; raw data obtained by monitoring the absorbance of the supernatant solution in the range of 250–400 nm for 90 min (a number of 19 spectra were recorded every 5 min, starting from 0 min—the lowest spectra—to 90 min—the highest spectra). Figure S13: Superimposed UV–Vis spectra for the controlled release studies on the transdermal pharmaceutical formulation based on β-CD/*M. chamomilla* leaf extract complex in ethanol 20% solution; raw data obtained by monitoring the absorbance of the supernatant solution in the range of 250–400 nm for 90 min (a number of 19 spectra were recorded every 5 min, starting from 0 min—the lowest spectra—to 90 min—the highest spectra). Figure S14: Superimposed UV–Vis spectra for the controlled release studies on the transdermal pharmaceutical formulation based on β-CD/*M. chamomilla* leaf extract complex in ethanol 60% solution; raw data obtained by monitoring the absorbance of the supernatant solution in the range of 250–400 nm for 90 min (a number of 19 spectra were recorded every 5 min, starting from 0 min—the lowest spectra—to 90 min—the highest spectra). Figure S15: Superimposed UV–Vis spectra for the controlled release studies on the transdermal pharmaceutical formulation based on β-CD/*M. chamomilla* leaf extract complex in ethanol 96% solution; raw data obtained by monitoring the absorbance of the supernatant solution in the range of 250–400 nm for 90 min (a number of 19 spectra were recorded every 5 min, starting from 0 min—the lowest spectra—to 90 min—the highest spectra). Figure S16: Superimposed UV–Vis spectra for the controlled release studies on the transdermal pharmaceutical formulation based on β-CD/*M. chamomilla* root extract complex in saline solution; raw data obtained by monitoring the absorbance of the supernatant solution in the range of 250–400 nm for 90 min (a number of 19 spectra were recorded every 5 min, starting from 0 min—the lowest spectra—to 90 min—the highest spectra). Figure S17: Superimposed UV–Vis spectra for the controlled release studies on the transdermal pharmaceutical formulation based on β-CD/*M. chamomilla* root extract complex in ethanol 20% solution; raw data obtained by monitoring the absorbance of the supernatant solution in the range of 250–400 nm for 90 min (a number of 19 spectra were recorded every 5 min, starting from 0 min—the lowest spectra—to 90 min—the highest spectra). Figure S18: Superimposed UV–Vis spectra for the controlled release studies on the transdermal pharmaceutical formulation based on β-CD/*M. chamomilla* root extract complex in ethanol 60% solution; raw data obtained by monitoring the absorbance of the supernatant solution in the range of 250–400 nm for 90 min (a number of 19 spectra were recorded every 5 min, starting from 0 min—the lowest spectra—to 90 min—the highest spectra). Figure S19: Superimposed UV–Vis spectra for the controlled release studies on the transdermal pharmaceutical formulation based on β-CD/*M. chamomilla* root extract complex in ethanol 96% solution; raw data obtained by monitoring the absorbance of the supernatant solution in the range of 250–400 nm for 90 min (a number of 19 spectra were recorded every 5 min, starting from 0 min—the lowest spectra—to 90 min—the highest spectra). Figure S20: Superimposed UV–Vis spectra for the controlled release studies on the transdermal pharmaceutical formulation based on β-CD/*M. chamomilla* stem extract complex in saline solution; raw data obtained by monitoring the absorbance of the supernatant solution in the range of 250–400 nm for 90 min (a number of 19 spectra were recorded every 5 min, starting from 0 min—the lowest spectra—to 90 min—the highest spectra). Figure S21: Superimposed UV–Vis spectra for the controlled release studies on the transdermal pharmaceutical formulation based on β-CD/*M. chamomilla* stem extract complex in ethanol 20% solution; raw data obtained by monitoring the absorbance of the supernatant solution in the range of 250–400 nm for 90 min (a number of 19 spectra were recorded every 5 min, starting from 0 min—the lowest spectra—to 90 min—the highest spectra). Figure S22: Superimposed UV–Vis spectra for the controlled release studies on the transdermal pharmaceutical formulation based on β-CD/*M. chamomilla* stem extract complex in ethanol 60% solution; raw data obtained by monitoring the absorbance of the supernatant solution in the range of 250–400 nm for 90 min (a number of 19 spectra were recorded every 5 min, starting from 0 min—the lowest spectra—to 90 min—the highest spectra). Figure S23: Superimposed UV–Vis spectra for the controlled release studies on the transdermal pharmaceutical formulation based on β-CD/*M. chamomilla* stem extract complex in ethanol 96% solution; raw data obtained by monitoring the absorbance of the supernatant solution in the range of 250–400 nm for 90 min (a number of 19 spectra were recorded every 5 min, starting from 0 min—the lowest spectra—to 90 min—the highest spectra). Figure S24: Superimposed UV–Vis spectra for the controlled release studies on the transdermal pharmaceutical formulation based on β-CD/*S. marianum* seed extract complex in saline solution; raw data obtained by monitoring the absorbance of the supernatant solution in the range of 250–400 nm for 90 min (a number of 19 spectra were recorded every 5 min, starting from 0 min—the lowest spectra—to 90 min—the highest spectra). Figure S25: Superimposed UV–Vis spectra for the controlled release studies on the transdermal pharmaceutical formulation based on β-CD/*S. marianum* seed extract complex in ethanol 20% solution; raw data obtained by monitoring the absorbance of the supernatant solution in the range of 250–400 nm for 90 min (a number of 19 spectra were recorded every 5 min, starting from 0 min—the lowest spectra—to 90 min—the highest spectra). Figure S26: Superimposed UV–Vis spectra for the controlled release studies on the transdermal pharmaceutical formulation based on β-CD/*S. marianum* seed extract complex in ethanol 60% solution; raw data obtained by monitoring the absorbance of the supernatant solution in the range of 250–400 nm for 90 min (a number of 19 spectra were recorded every 5 min, starting from 0 min—the lowest spectra—to 90 min—the highest spectra). Figure S27: Superimposed UV–Vis spectra for the controlled release studies on the transdermal pharmaceutical formulation based on β-CD/*S. marianum* seed extract complex in ethanol 96% solution; raw data obtained by monitoring the absorbance of the supernatant solution in the range of 250–400 nm for 90 min (a number of 19 spectra were recorded every 5 min, starting from 0 min—the lowest spectra—to 90 min—the highest spectra). Figure S28: Superimposed UV–Vis spectra for the controlled release studies on the transdermal pharmaceutical formulation based on β-CD/silibinin diastereomer mixture complex in saline solution; raw data obtained by monitoring the absorbance of the supernatant solution in the range of 250–400 nm for 90 min (a number of 19 spectra were recorded every 5 min, starting from 0 min—the lowest spectra—to 90 min—the highest spectra). Figure S29: Superimposed UV–Vis spectra for the controlled release studies on the transdermal pharmaceutical formulation based on β-CD/silibinin diastereomer mixture complex in ethanol 20% solution; raw data obtained by monitoring the absorbance of the supernatant solution in the range of 250–400 nm for 90 min (a number of 19 spectra were recorded every 5 min, starting from 0 min—the lowest spectra—to 90 min—the highest spectra). Figure S30: Superimposed UV–Vis spectra for the controlled release studies on the transdermal pharmaceutical formulation based on β-CD/silibinin diastereomer mixture complex in ethanol 60% solution; raw data obtained by monitoring the absorbance of the supernatant solution in the range of 250–400 nm for 90 min (a number of 19 spectra were recorded every 5 min, starting from 0 min—the lowest spectra—to 90 min—the highest spectra). Figure S31: Superimposed UV–Vis spectra for the controlled release studies on the transdermal pharmaceutical formulation based on β-CD/silibinin diastereomer mixture complex in ethanol 96% solution; raw data obtained by monitoring the absorbance of the supernatant solution in the range of 250–400 nm for 90 min (a number of 19 spectra were recorded every 5 min, starting from 0 min—the lowest spectra—to 90 min—the highest spectra). Figure S32: Superimposed UV–Vis spectra for the controlled release studies on the transdermal pharmaceutical formulation based on β-CD/silymarin mixture complex in saline solution; raw data obtained by monitoring the absorbance of the supernatant solution in the range of 250–400 nm for 90 min (a number of 19 spectra were recorded every 5 min, starting from 0 min—the lowest spectra—to 90 min—the highest spectra). Figure S33: Superimposed UV–Vis spectra for the controlled release studies on the transdermal pharmaceutical formulation based on β-CD/silymarin mixture complex in ethanol 20% solution; raw data obtained by monitoring the absorbance of the supernatant solution in the range of 250–400 nm for 90 min (a number of 19 spectra were recorded every 5 min, starting from 0 min—the lowest spectra—to 90 min—the highest spectra). Figure S34: Superimposed UV–Vis spectra for the controlled release studies on the transdermal pharmaceutical formulation based on β-CD/silymarin mixture complex in ethanol 60% solution; raw data obtained by monitoring the absorbance of the supernatant solution in the range of 250–400 nm for 90 min (a number of 19 spectra were recorded every 5 min, starting from 0 min—the lowest spectra—to 90 min—the highest spectra). Figure S35: Superimposed UV–Vis spectra for the controlled release studies on the transdermal pharmaceutical formulation based on β-CD/silymarin mixture complex in ethanol 96% solution; raw data obtained by monitoring the absorbance of the supernatant solution in the range of 250–400 nm for 90 min (a number of 19 spectra were recorded every 5 min, starting from 0 min—the lowest spectra—to 90 min—the highest spectra). Figure S36: Controlled release of antioxidant compounds from the β-cyclodextrin/*M. chamomilla* flower and leaf extract complexes (expressed as chrysin, μg/mL) in ethanol 20% (brown), ethanol 60% (green), and ethanol 96% (bleu); number of replicate determinations *n* = 2; error bars were determined from standard errors using a coefficient of ±0.95. Figure S37: Controlled release of antioxidant compounds from the β-cyclodextrin/*M. chamomilla* root and stem extract complexes (expressed as rutin, μg/mL) in ethanol 20% (brown), ethanol 60% (green), and ethanol 96% (bleu); number of replicate determinations *n* = 2; error bars were determined from standard errors using a coefficient of ±0.95. Figure S38: Controlled release of antioxidant compounds from the β-cyclodextrin/*M. chamomilla* root and stem extract complexes (expressed as chrysin, μg/mL) in ethanol 20% (brown), ethanol 60% (green), and ethanol 96% (bleu); number of replicate determinations *n* = 2; error bars were determined from standard errors using a coefficient of ±0.95. Figure S39: Controlled release of antioxidant compounds (expressed as chrysin, μg/mL) from the transdermal pharmaceutical formulations containing β-cyclodextrin/*M. chamomilla* flower and leaf extract complexes in saline solution (brown), ethanol 20% (green), ethanol 60% (bleu), and ethanol 96% (black); number of replicate determinations *n* = 2; error bars were determined from standard errors using a coefficient of ±0.95. Figure S40: Controlled release of antioxidant compounds (expressed as chrysin, μg/mL) from the transdermal pharmaceutical formulations containing β-cyclodextrin/*M. chamomilla* root and stem extract complexes in saline solution (brown), ethanol 20% (green), ethanol 60% (bleu), and ethanol 96% (black); number of replicate determinations *n* = 2; error bars were determined from standard errors using a coefficient of ±0.95. Figure S41: Controlled release of antioxidant compounds (expressed as silibinin, μg/mL) from the transdermal pharmaceutical formulations containing β-cyclodextrin/silymarin complexes in saline solution (brown), ethanol 20% (green), ethanol 60% (bleu), and ethanol 96% (black); number of replicate determinations *n* = 2; error bars were determined from standard errors using a coefficient of ±0.95. Table S6: Korsmeyer–Peppas modeling (*M_t_*/*M_∞_* = *k(_KP)_*·*t^n^*) of the controlled release of antioxidants from β-cyclodextrin complexes. *M_t_* and *M_∞_* stand for the amount (mg) of antioxidant released from the complex at the time *t* (s) and the total amount, respectively. The Korsmeyer–Peppas kinetic constant, *k*_(*KP*)_ (s^−n^), and the diffusional exponent, *n*, were determined by fitting the experimental data (least squares approximation). Codes for complexes are: *bCD_MChXY*—β-cyclodextrin/*M. chamomilla* L. extract; *bCD_SMaY*—β-cyclodextrin/*S. marianum* L. extract; *X* stands for the plant part, *X* = *F*—flowers, *L*—leaves, *R*—roots or *S*—stems; *bCD_Sb/SmY*—β-cyclodextrin/silibinin or β-cyclodextrin/silymarin complexes; *Y* stands for the concentration of ethanol solution used for controlled release (20, 60, and 96%, *v*/*v*). Table S7: Korsmeyer–Peppas modeling (*M_t_*/*M_∞_* = *k(_KP)_*·*t^n^*) of the controlled release of antioxidants from transdermal pharmaceutical formulations based on β-cyclodextrin complexes. *M_t_* and *M_∞_* stand for the amount (mg) of antioxidant released from the transdermal pharmaceutical formulation at the time *t* (s) and the total amount, respectively. The Korsmeyer–Peppas kinetic constant, *k*_(*KP*)_ (s^−n^), and the diffusional exponent, *n*, were determined by fitting the experimental data (least squares approximation). Codes for the transdermal pharmaceutical formulations based on cyclodextrin complexes are: *bCD_MChX_TFY*—transdermal pharmaceutical formulation based on β-cyclodextrin/*M. chamomilla* L. extract; *bCD_SMa_TFY*—transdermal pharmaceutical formulation based on β-cyclodextrin/*S. marianum* L. extract; *X* stands for the plant part, *X* = *F*—flowers, *L*—leaves, *R*—roots, or *S*—stems; *bCD_Sb/Sm_TFY*—transdermal pharmaceutical formulation based on β-cyclodextrin/silibinin or β-cyclodextrin/silymarin complexes; *Y* stands for saline solution (“00”) or the concentration of ethanol solution used for controlled release (20, 60, and 96%, *v*/*v*).
