# Supplementary material for: Estimation of the Controlled Release of Antioxidants from β-Cyclodextrin/Chamomile (Matricaria chamomilla L.) or Milk Thistle (Silybum marianum L.), Asteraceae, Hydrophilic Extract Complexes through the Fast and Cheap Spectrophotometric Technique
Source: Plants (Basel). 2023 Jun 17;12(12):2352. doi: 10.3390/plants12122352 (PMC10303029; doi:10.3390/plants12122352)
Supplement: Supplementary file 1 [file plants-12-02352-s001.zip › plants-2435815-supplementary.pdf]

plants

Article

Supplementary material

Estimation of the controlled release of antioxidants from β-cyclodextrin/chamomile (*Matricaria chamomilla* L.) or milk thistle (*Silybum marianum* L.), Asteraceae, hydrophilic extract complexes through the fast and cheap spectrophotometric technique

Adina Horablaga [1], Alina Şibu (Ciobanu) [2], Corina Iuliana Megyesi [3], Dina Gligor (Pane) [2], Gabriel Stelian Bujancă [4], Ariana Bianca Velciov [3], Florica Emilia Morariu [5,*], Daniel Ioan Hădărugă [2,6], Corina Dana Mişcă [3,*], Nicoleta Gabriela Hădărugă [2,3,*]

[1] Department of Sustainable Development and Environmental Engineering, University of Life Sciences "King Mihai I" from Timişoara, Calea Aradului 119, 300645-Timişoara, Romania; ahorablaga@yahoo.com; adinahorablaga@usvt.ro (A.H.)
[2] Doctoral School "Engineering of Vegetable and Animal Resources", University of Life Sciences "King Mihai I" from Timişoara, Calea Aradului 119, 300645-Timişoara, Romania; ciobanualina1966@yahoo.com (A.S.C.); gligor_dina_bihor@yahoo.com (D.G.P.)
[3] Department of Food Science, University of Life Sciences "King Mihai I" from Timişoara, Calea Aradului 119, 300645-Timişoara, Romania; corina.megyesi@usvt.ro; cor_costescu@yahoo.com (C.I.M.); arianavelciov@usvt.ro; ariana.velciov@yahoo.com (A.B.V.) nicoletahadaruga@usvt.ro; nico_hadaruga@yahoo.com (N.G.H.)
[4] Department of Food Control, University of Life Sciences "King Mihai I" from Timişoara, Calea Aradului 119, 300645-Timişoara, Romania; gabrielbujanca@yahoo.com (G.S.B.)
[5] Department of Biotechnologies, University of Life Sciences "King Mihai I" from Timişoara, Calea Aradului 119, 300645-Timişoara, Romania; florica.morariu@animalsci-tm.ro (F.E.M.)
[6] Department of Applied Chemistry, Organic and Natural Compounds Engineering, Polytechnic University of Timişoara, Carol Telbisz 6, 300001 – Timişoara, Romania; daniel.hadaruga@upt.ro (D.I.H.)
* Correspondence: nico_hadaruga@yahoo.com (N.G.H.); florica.morariu@animalsci-tm.ro (F.E.M.); miscacorina@gmail.com (C.D.M); Tel.: +40-256-277-423

Citation: Horablaga, A.; Şibu (Ciobanu), A.; Megyesi, C.I.; Gligor (Pane), D.; Bujancă, G.S.; Velciov, A.B.; Morariu, F.E.; Hădărugă, D.I.; Mişcă, C.D.; Hădărugă, N.G. Estimation of the controlled release of antioxidants from β-cyclodextrin/chamomile (*Matricaria chamomilla* L.) or milk thistle (*Silybum marianum* L.), Asteraceae, hydrophilic extract complexes through the fast and cheap spectrophotometric technique. *Plants* **2023**, *12*, 2352. https://doi.org/10.3390/plants12122352

Copyright: © 2023 by the authors. Submitted for possible open access publication under the terms and conditions of the Creative Commons Attribution (CC BY) license (https://creativecommons.org/licenses/by/4.0/).

1. Obtaining of *Matricaria chamomilla* and *Silybum marianum* extracts

Table S1. Raw data and results for the obtaining of *M. matricaria* extracts

Code	Description	Sample mass (g)	Solvent volume (mL)	Final extract volume (mL)
MChF20	*M. chamomilla* (flowers) extract using ethanol 20%	5.31	70.0	30.0
MChL20	*M. chamomilla* (leaves) extract using ethanol 20%	5.53	70.0	27.0
MChR20	*M. chamomilla* (roots) extract using ethanol 20%	5.37	70.0	29.5
MChS20	*M. chamomilla* (stems) extract using ethanol 20%	5.20	70.0	28.5
MChF40	*M. chamomilla* (flowers) extract using ethanol 40%	5.40	70.0	30.0
MChL40	*M. chamomilla* (leaves) extract using ethanol 40%	5.17	70.0	30.5
MChR40	*M. chamomilla* (roots) extract using ethanol 40%	5.35	70.0	30.0
MChS40	*M. chamomilla* (stems) extract using ethanol 40%	5.28	70.0	29.0
MChF60	*M. chamomilla* (flowers) extract using ethanol 60%	5.16	70.0	30.0
MChL60	*M. chamomilla* (leaves) extract using ethanol 60%	5.32	70.0	29.5
MChR60	*M. chamomilla* (roots) extract using ethanol 60%	5.02	70.0	30.5
MChS60	*M. chamomilla* (stems) extract using ethanol 60%	5.48	70.0	30.0
MChF80	*M. chamomilla* (flowers) extract using ethanol 80%	5.32	70.0	30.0
MChL80	*M. chamomilla* (leaves) extract using ethanol 80%	5.11	70.0	29.5
MChR80	*M. chamomilla* (roots) extract using ethanol 80%	5.17	70.0	30.0
MChS80	*M. chamomilla* (stems) extract using ethanol 80%	5.61	70.0	28.0
MChF96	*M. chamomilla* (flowers) extract using ethanol 96%	5.40	70.0	30.0
MChL96	*M. chamomilla* (leaves) extract using ethanol 96%	5.13	70.0	25.0
MChR96	*M. chamomilla* (roots) extract using ethanol 96%	3.85	70.0	25.0
MChS96	*M. chamomilla* (stems) extract using ethanol 96%	5.42	70.0	28.5

Table S2. Raw data and results for the obtaining of *S. marianum* extracts

Code	Description	Sample mass (g)	Solvent volume (mL)	Final extract volume (mL)
SMa20	*S. marianum* (seeds) extract using ethanol 20%	5.51	70.0	28.5
SMa40	*S. marianum* (seeds) extract using ethanol 40%	5.52	70.0	29.5
SMa60	*S. marianum* (seeds) extract using ethanol 60%	5.57	70.0	30.0
SMa80	*S. marianum* (seeds) extract using ethanol 80%	5.26	70.0	25.5
SMa96	*S. marianum* (seeds) extract using ethanol 96%	5.46	70.0	26.5

Table S3. Results obtained for the flavonoid content (as chrysin or rutin, mg/g FW) of *M. chamomilla* flowers, leaves, roots and stems using ethanol of various concentrations (*v*/*v*) for extraction.

Code	EtOH 20%	EtOH 40%	EtOH 60%	EtOH 80%	EtOH 96%
MChF (chrysin)	8.03*	5.63*	1.77*	4.80*	7.27*
MChF (rutin)	23.41*	16.40*	5.15*	13.98*	21.21*
MChL (chrysin)	5.20±2.90	4.50±1.42	4.43±1.54	3.40±0.81	8.88±5.73
MChL (rutin)	15.16±8.45	13.13±4.14	12.91±4.50	9.92±2.26	25.89±16.69
MChR (chrysin)	1.04±0.27	0.82±0.01	3.65±1.82	1.02±0.18	2.92*
MChR (rutin)	3.05±0.78	2.37±0.04	10.65±5.32	2.97±0.51	8.50*
MChS (chrysin)	2.42±1.87	2.22±1.43	1.89±0.69	1.25±0.24	2.58±2.19
MChS (rutin)	7.04±5.44	6.46±4.17	5.50±2.01	3.65±0.69	7.51±6.39

* single sample (mean values determined from nine diluted extracts)

Table S4. Results obtained for the silibinin content (based on both silibinin diastereomer mixture and silymarin mixture, mg/g FW) of *S. marianum* using ethanol of various concentrations (*v*/*v*) for extraction.

Code	EtOH 20%	EtOH 40%	EtOH 60%	EtOH 80%	EtOH 96%
SMa_Sb (silibinin diastereomers)	3.00±0.76	7.65±4.60	6.47±3.54	6.13±1.62	6.69±3.77
SMa_Sm (silymarin mixture)	3.53±0.90	9.01±5.41	7.62±4.17	7.21±1.90	7.88±4.43

2. Obtaining of β-cyclodextrin / vegetable extract complexes

Table S5. Results obtained for β-cyclodextrin/vegetable extract or β-cyclodextrin/flavonolignan complexes (codes for complexes: *bCD_MChX* – β-cyclodextrin/*M. chamomilla* L. extract; *bCD_SMaX* – β-cyclodextrin/*S. marianum* L. extract; *X* stands for the plant part, *X* = *F* – flowers, *L* – leaves, *R* – roots or *S* – stems; *bCD_Sb*/*Sm* – β-cyclodextrin/silibinin or β-cyclodextrin/silymarin complexes).

Code	β-Cyclodextrin mass (g)	*Extract volume (mL)*	*Flavonolignan mass (g)*	*Water volume (mL)*	*Yield (%)*
bCD_MChF	0.671	4	-	4	58.81
bCD_MChL	0.672	4	-	4	55.71
bCD_MChR	0.670	4	-	4	61.87
bCD_MChS	0.671	4	-	4	60.15
bCD_SMa	0.670	4	-	4	75.72
bCD_Sb	0.671	-	0.242	4	87.03
bCD_Sm	0.670	-	0.241	4	86.47

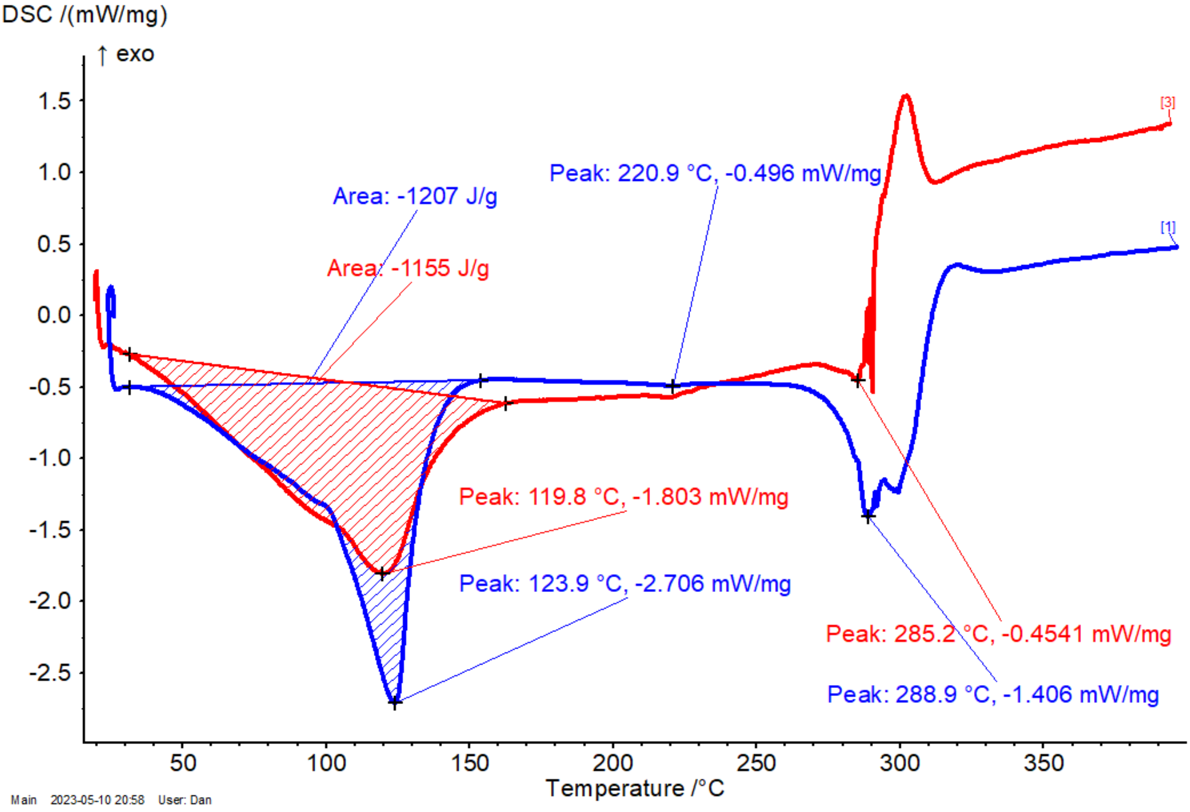

Figure S1. DSC results for the β-CD/*M. chamomilla* leaf extract complex (red) and β-CD hydrate (blue); the DSC conditions were set from 20 to 400 °C, with a heating rate of 4 °C/min, under nitrogen (purge and flow).

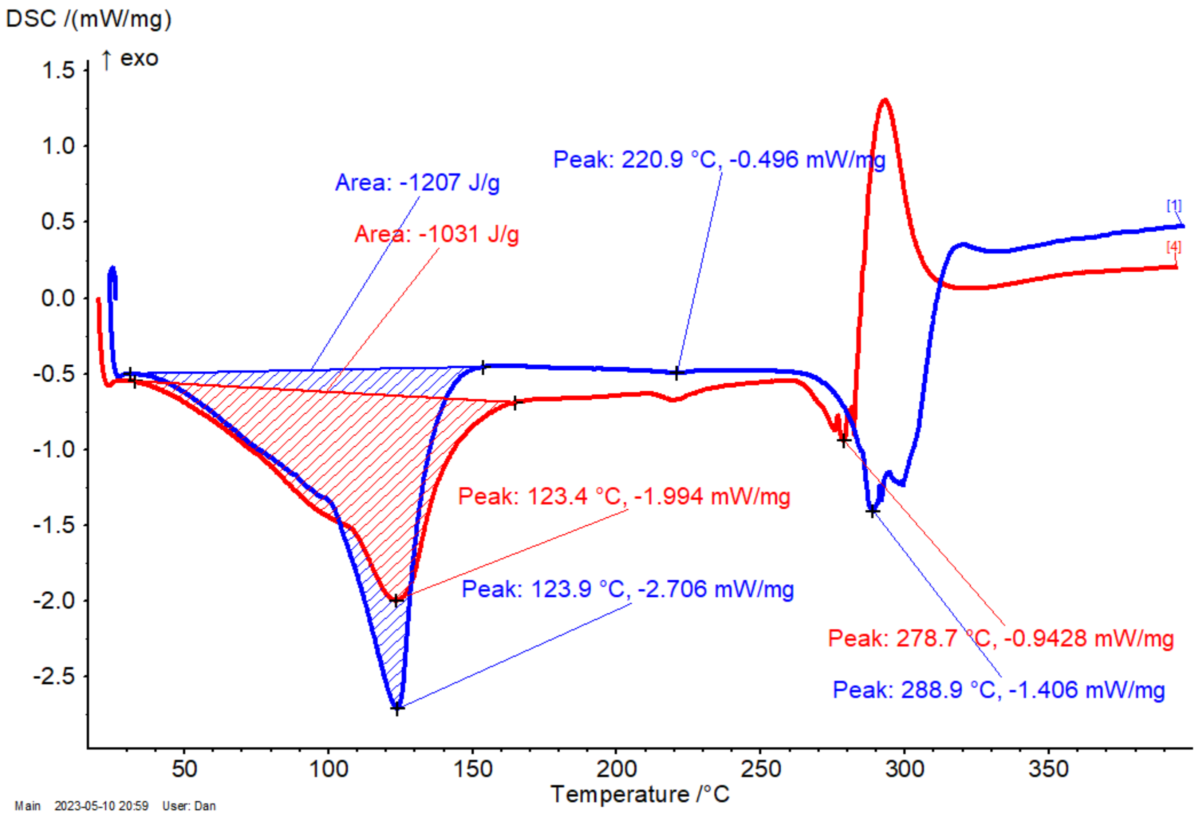

Figure S2. DSC results for the β-CD/*M. chamomilla* root extract complex (red) and β-CD hydrate (blue); the DSC conditions were set from 20 to 400 °C, with a heating rate of 4 °C/min, under nitrogen (purge and flow).

3. Controlled release of antioxidants from crystalline β-CD/vegetable extract complexes and transdermal pharmaceutical formulations containing β-CD/vegetable extract complexes

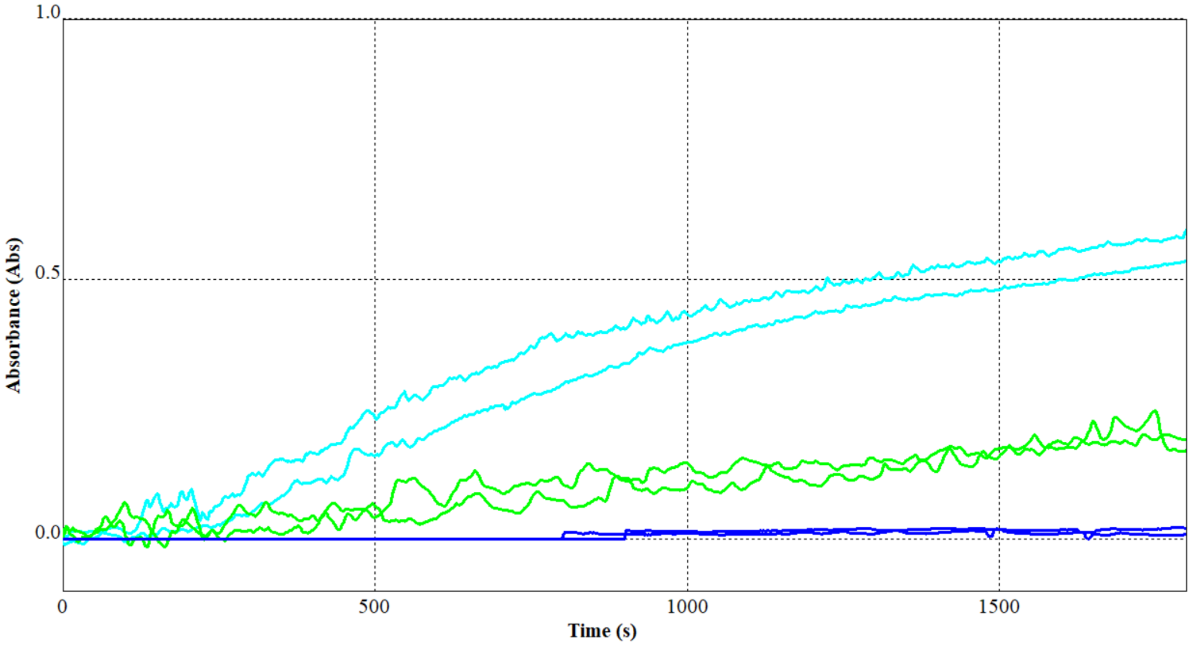

Figure S3. Superimposed *Absorbance versus Time* (s) plots for the controlled release studies on β-CD/*M. chamomilla* flower and leaf extracts in ethanol 20% (blue), ethanol 60% (green) and ethanol 96% (turquoise); raw data obtained by monitoring the absorbance of the supernatant solution at 322 nm for 30 min.

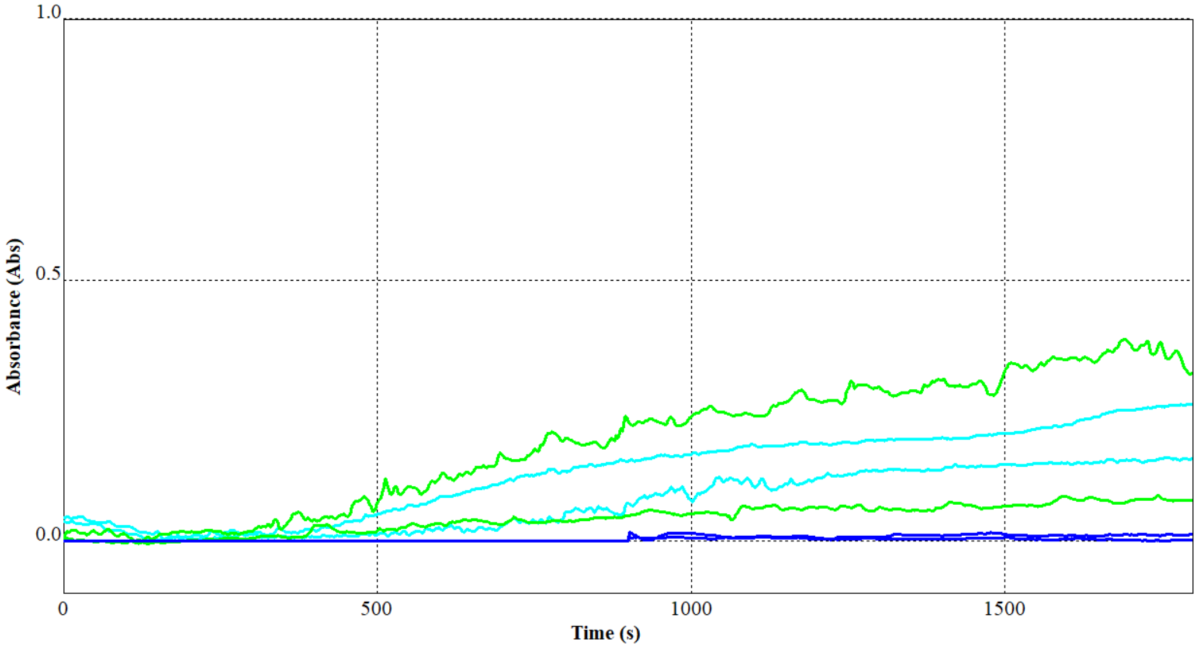

Figure S4. Superimposed *Absorbance versus Time* (s) plots for the controlled release studies on β-CD/*M. chamomilla* root and stem extracts in ethanol 20% (blue), ethanol 60% (green) and ethanol 96% (turquoise); raw data obtained by monitoring the absorbance of the supernatant solution at 322 nm for 30 min.

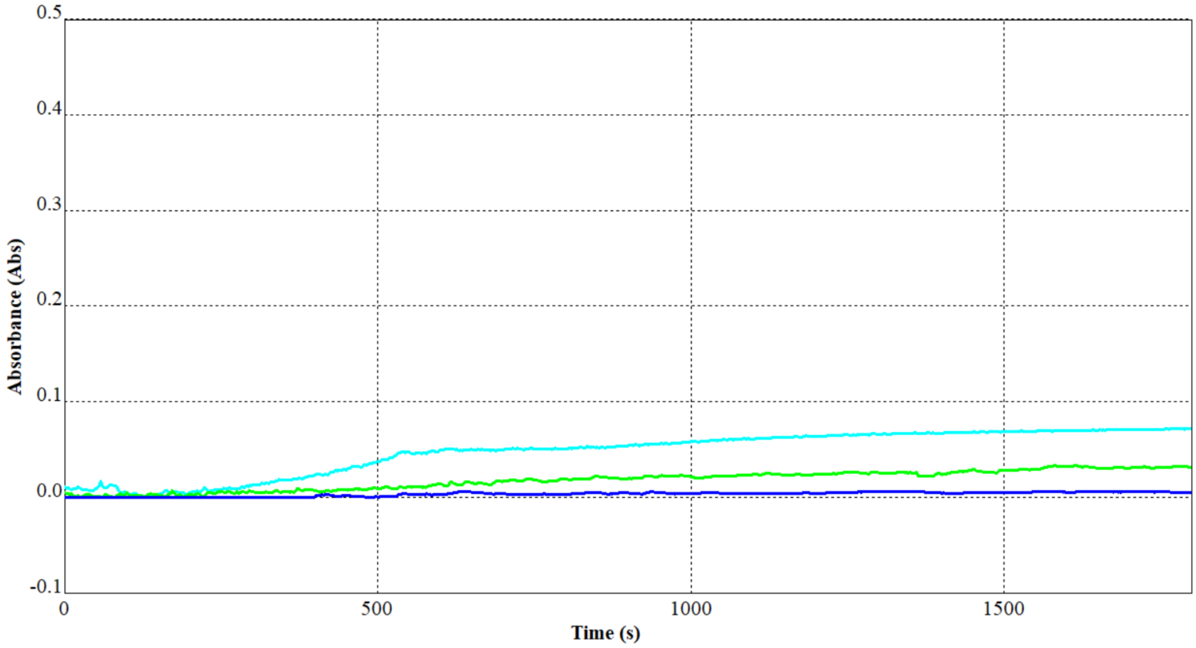

Figure S5. Superimposed *Absorbance versus Time* (s) plots for the controlled release studies on β-CD/*S. marianum* seed extracts in ethanol 20% (blue), ethanol 60% (green) and ethanol 96% (turquoise); raw data obtained by monitoring the absorbance of the supernatant solution at 288 nm for 30 min.

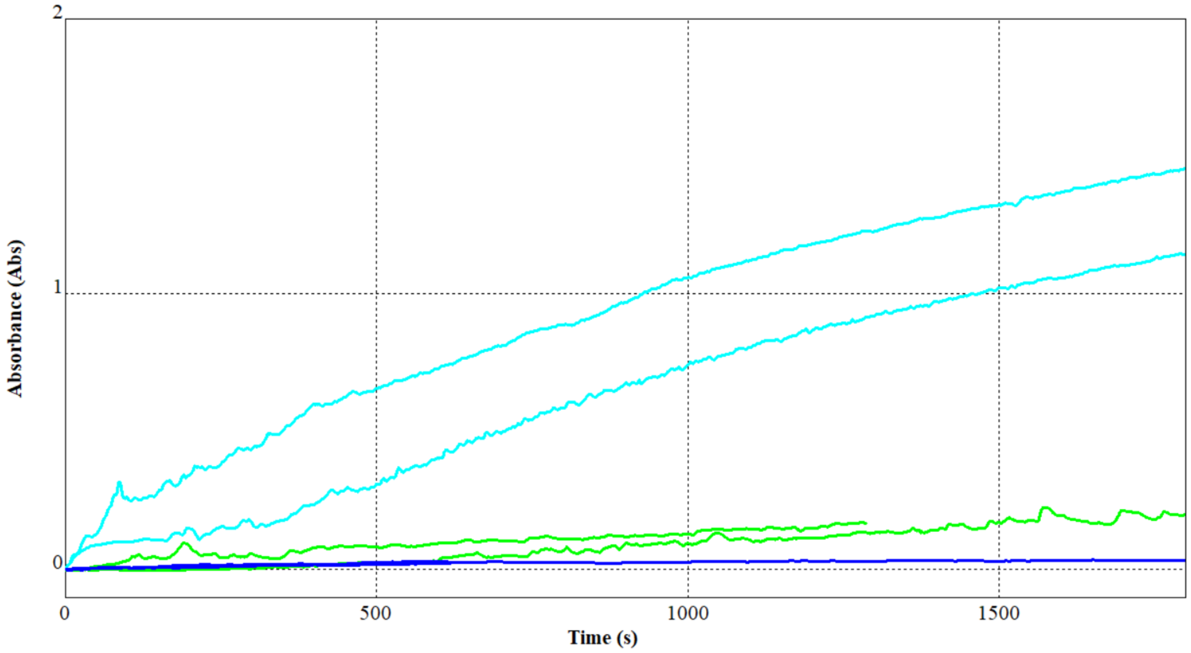

Figure S6. Superimposed *Absorbance versus Time* (s) plots for the controlled release studies on β-CD/silibinin diastereomer mixture complex in ethanol 20% (blue), ethanol 60% (green) and ethanol 96% (turquoise); raw data obtained by monitoring the absorbance of the supernatant solution at 288 nm for 30 min.

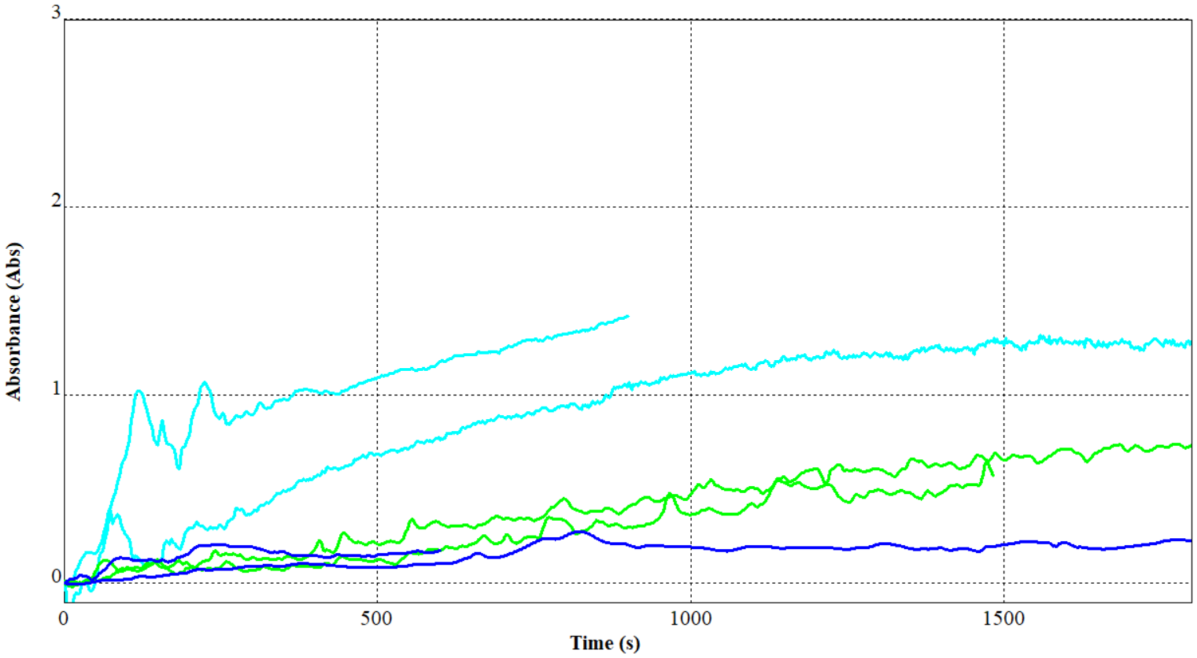

Figure S7. Superimposed *Absorbance versus Time* (s) plots for the controlled release studies on β-CD/silymarin standard mixture complex in ethanol 20% (blue), ethanol 60% (green) and ethanol 96% (turquoise); raw data obtained by monitoring the absorbance of the supernatant solution at 288 nm for 30 min.

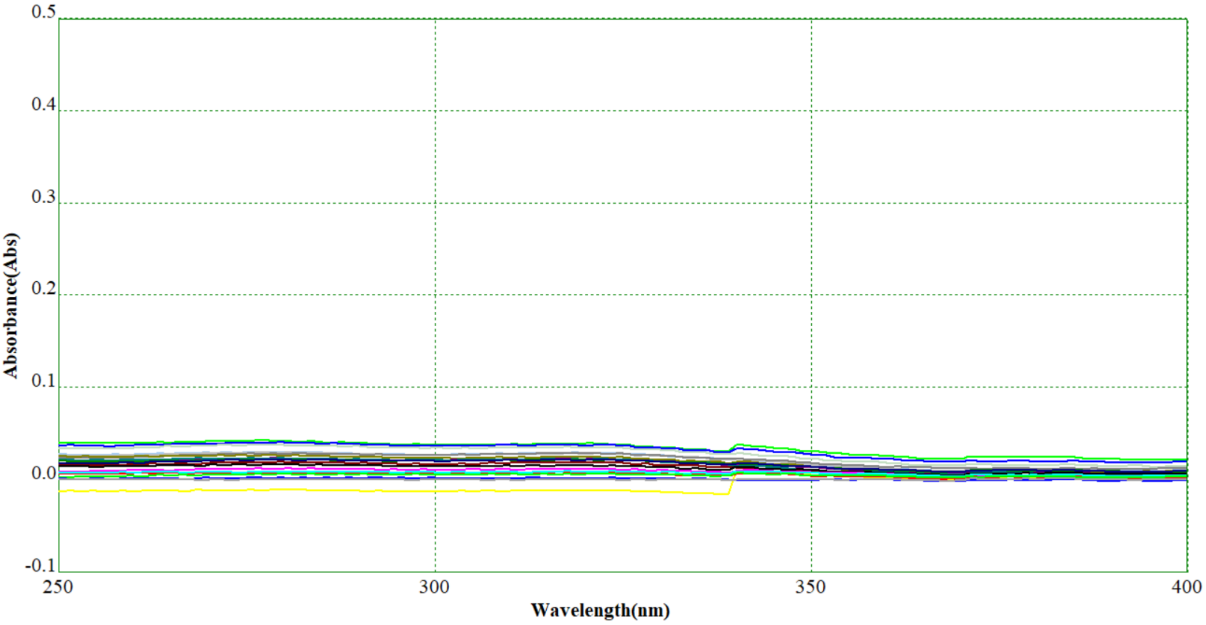

Figure S8. Superimposed UV-Vis spectra for the controlled release studies on the transdermal pharmaceutical formulation based on β-CD/*M. chamomilla* flower extract complex in saline solution; raw data obtained by monitoring the absorbance of the supernatant solution in the range of 250-400 nm for 90 min (a number of 19 spectra were recorded every 5 min, starting from 0 min – the lowest spectra to 90 min – the highest spectra).

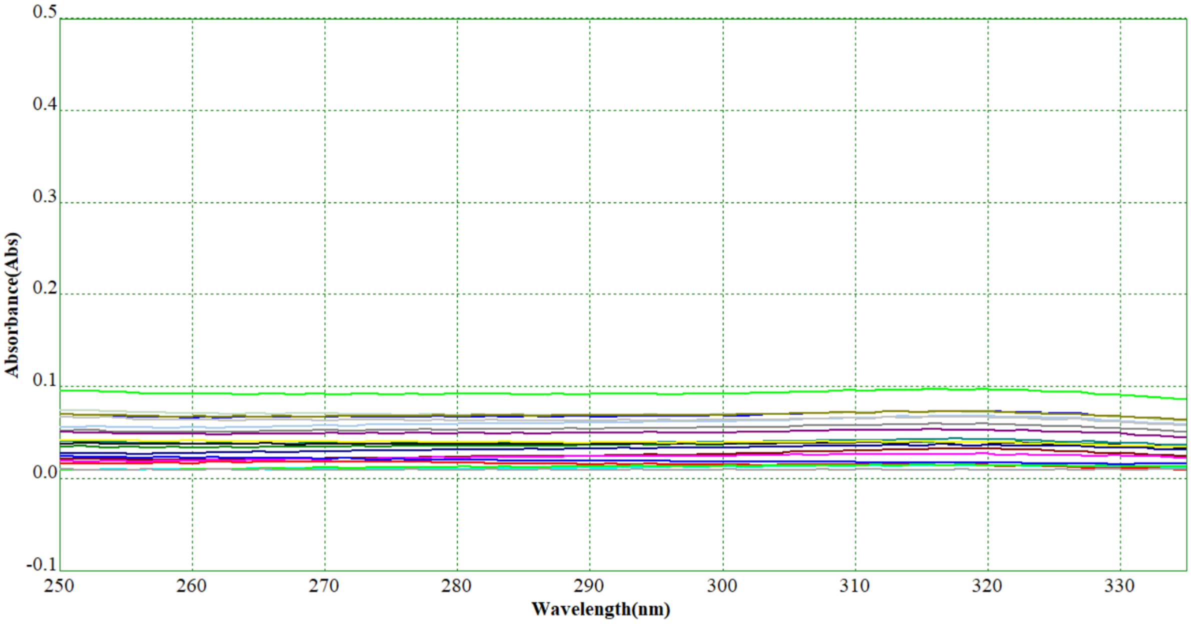

Figure S9. Superimposed UV-Vis spectra for the controlled release studies on the transdermal pharmaceutical formulation based on β-CD/*M. chamomilla* flower extract complex in ethanol 20% solution; raw data obtained by monitoring the absorbance of the supernatant solution in the range of 250-400 nm for 90 min (a number of 19 spectra were recorded every 5 min, starting from 0 min – the lowest spectra to 90 min – the highest spectra).

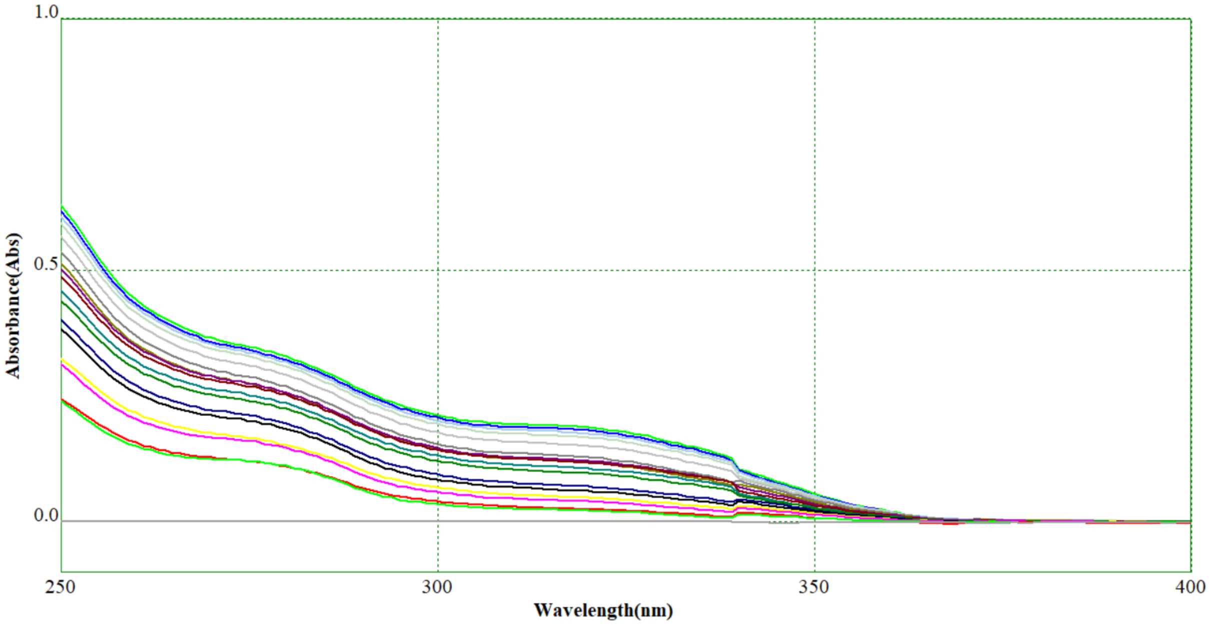

Figure S10. Superimposed UV-Vis spectra for the controlled release studies on the transdermal pharmaceutical formulation based on β-CD/*M. chamomilla* flower extract complex in ethanol 60% solution; raw data obtained by monitoring the absorbance of the supernatant solution in the range of 250-400 nm for 90 min (a number of 19 spectra were recorded every 5 min, starting from 0 min – the lowest spectra to 90 min – the highest spectra).

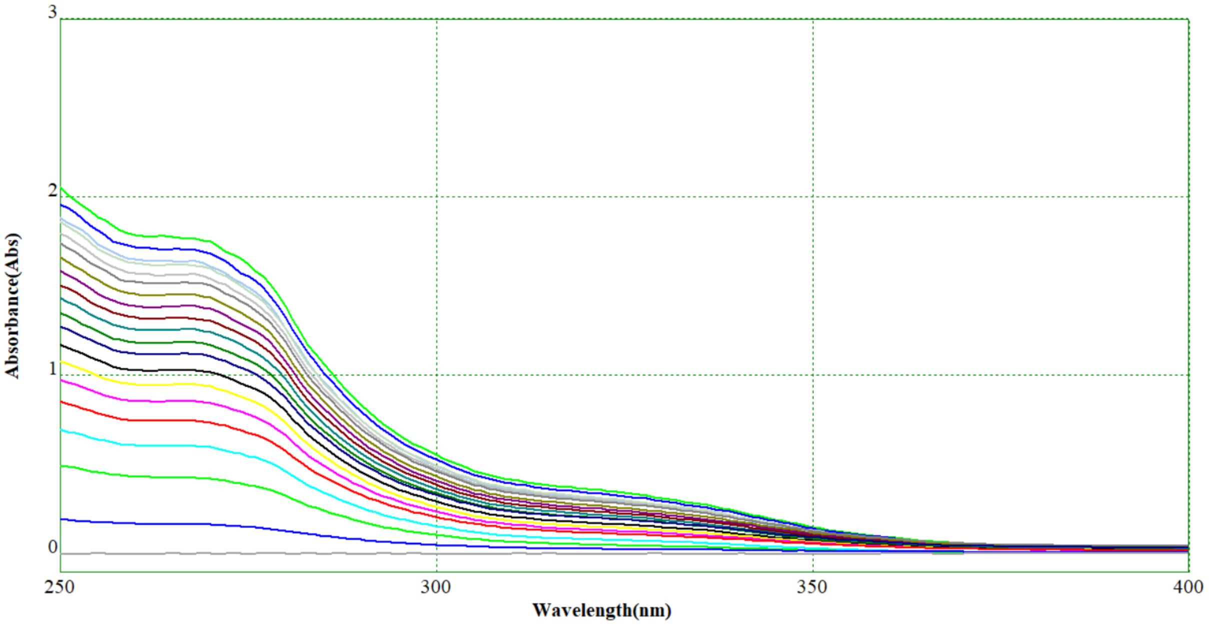

Figure S11. Superimposed UV-Vis spectra for the controlled release studies on the transdermal pharmaceutical formulation based on β-CD/*M. chamomilla* flower extract complex in ethanol 96% solution; raw data obtained by monitoring the absorbance of the supernatant solution in the range of 250-400 nm for 90 min (a number of 19 spectra were recorded every 5 min, starting from 0 min – the lowest spectra to 90 min – the highest spectra).

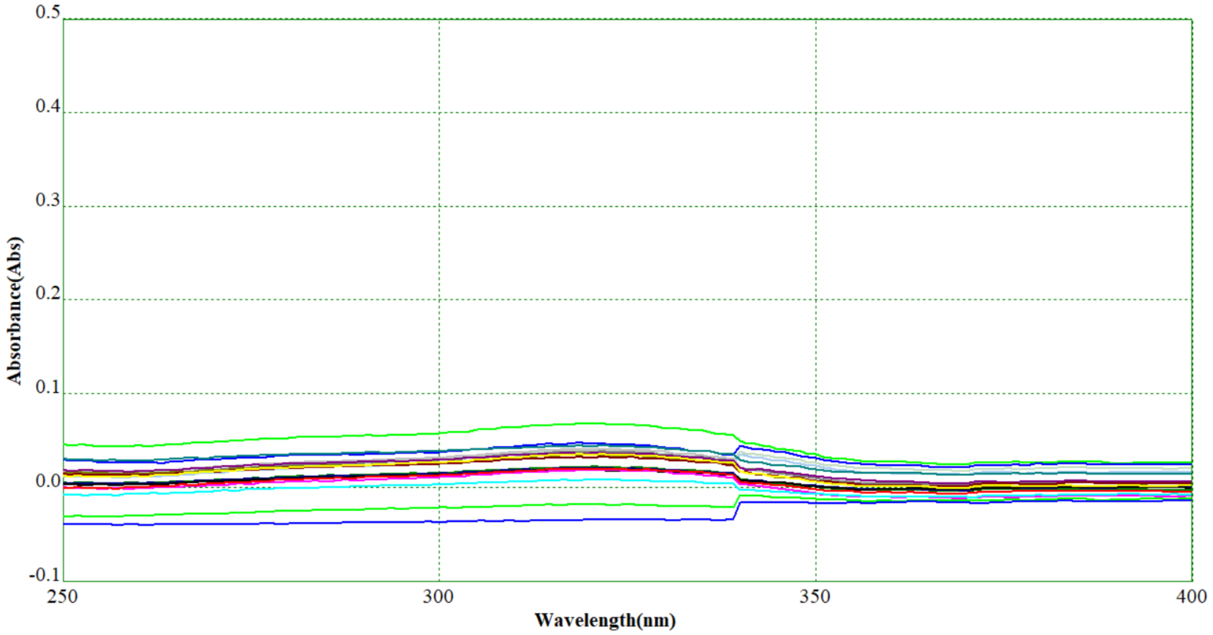

Figure S12. Superimposed UV-Vis spectra for the controlled release studies on the transdermal pharmaceutical formulation based on β-CD/*M. chamomilla* leaf extract complex in saline solution; raw data obtained by monitoring the absorbance of the supernatant solution in the range of 250-400 nm for 90 min (a number of 19 spectra were recorded every 5 min, starting from 0 min – the lowest spectra to 90 min – the highest spectra).

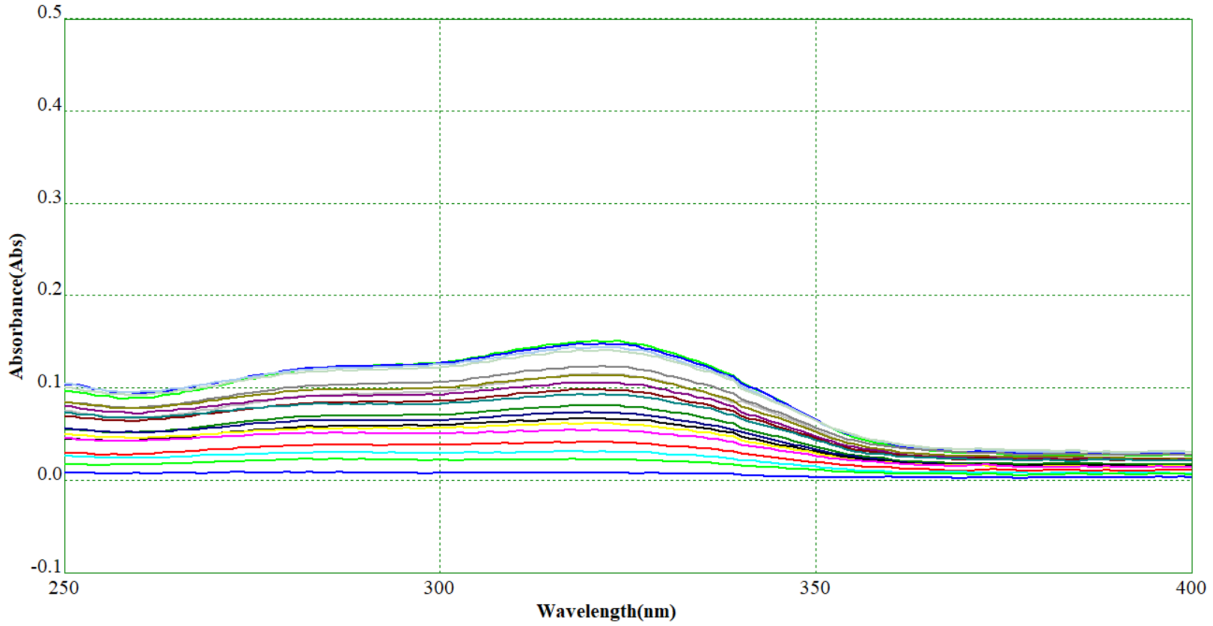

Figure S13. Superimposed UV-Vis spectra for the controlled release studies on the transdermal pharmaceutical formulation based on β-CD/*M. chamomilla* leaf extract complex in ethanol 20% solution; raw data obtained by monitoring the absorbance of the supernatant solution in the range of 250-400 nm for 90 min (a number of 19 spectra were recorded every 5 min, starting from 0 min – the lowest spectra to 90 min – the highest spectra).

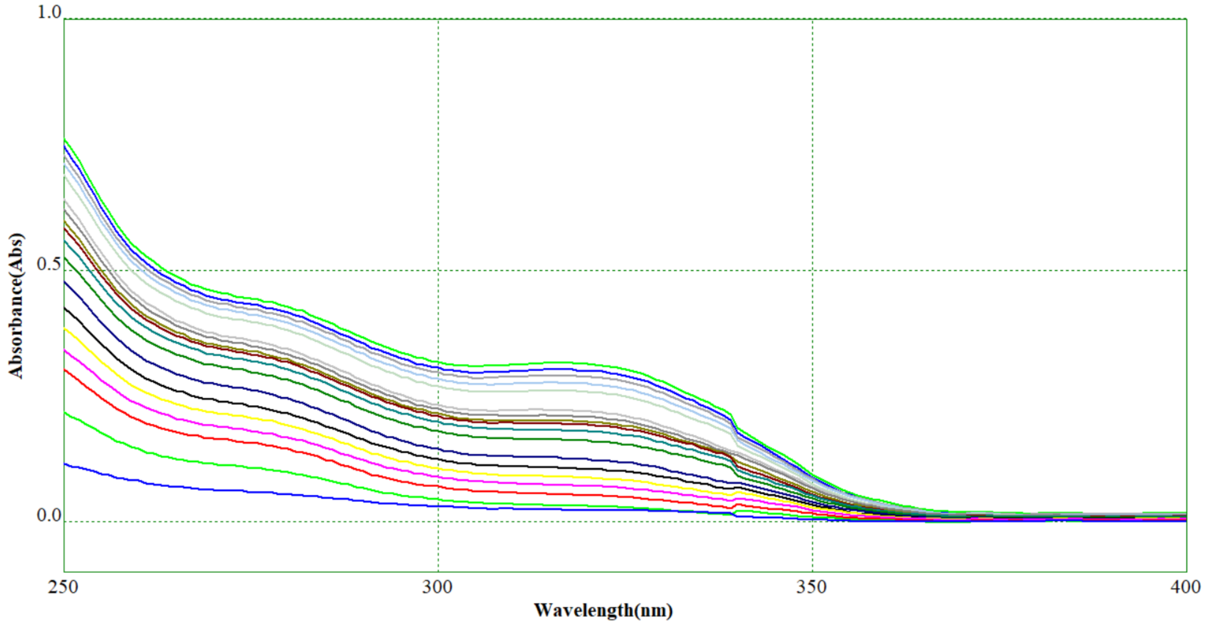

Figure S14. Superimposed UV-Vis spectra for the controlled release studies on the transdermal pharmaceutical formulation based on β-CD/*M. chamomilla* leaf extract complex in ethanol 60% solution; raw data obtained by monitoring the absorbance of the supernatant solution in the range of 250-400 nm for 90 min (a number of 19 spectra were recorded every 5 min, starting from 0 min – the lowest spectra to 90 min – the highest spectra).

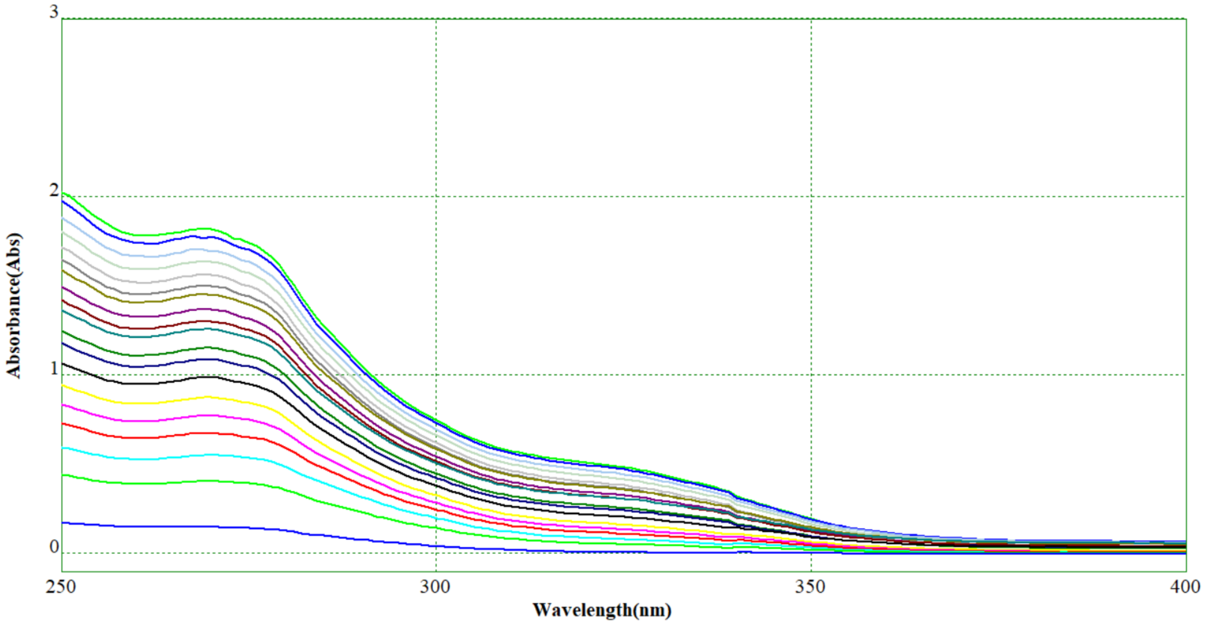

Figure S15. Superimposed UV-Vis spectra for the controlled release studies on the transdermal pharmaceutical formulation based on β-CD/*M. chamomilla* leaf extract complex in ethanol 96% solution; raw data obtained by monitoring the absorbance of the supernatant solution in the range of 250-400 nm for 90 min (a number of 19 spectra were recorded every 5 min, starting from 0 min – the lowest spectra to 90 min – the highest spectra).

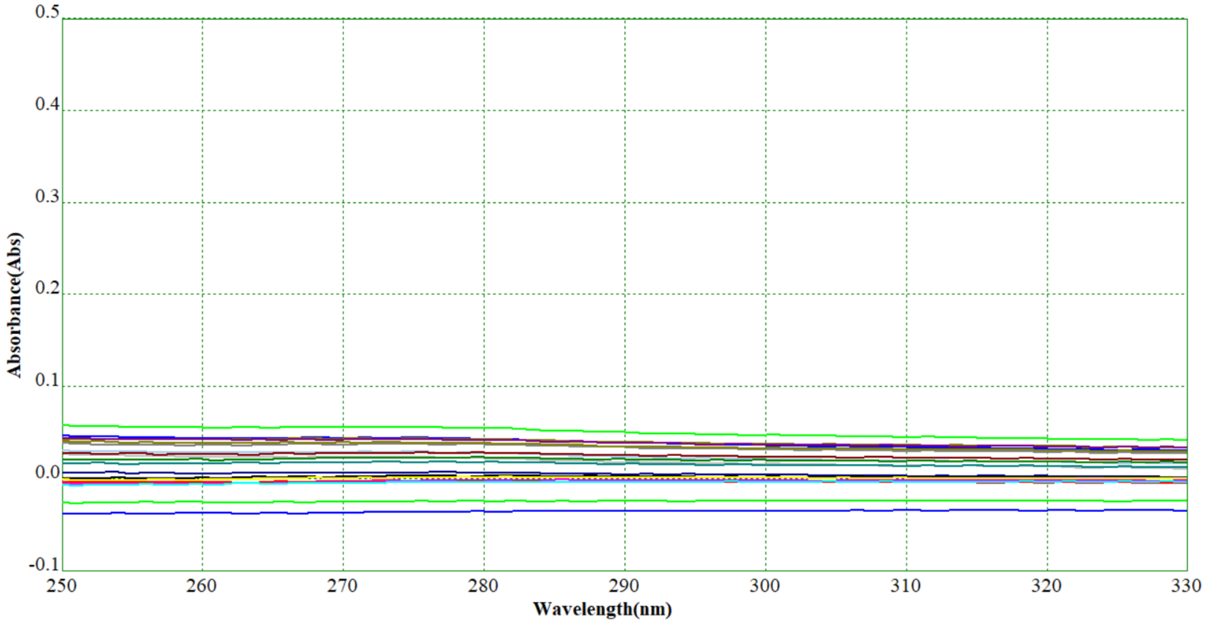

Figure S16. Superimposed UV-Vis spectra for the controlled release studies on the transdermal pharmaceutical formulation based on β-CD/*M. chamomilla* root extract complex in saline solution; raw data obtained by monitoring the absorbance of the supernatant solution in the range of 250-400 nm for 90 min (a number of 19 spectra were recorded every 5 min, starting from 0 min – the lowest spectra to 90 min – the highest spectra).

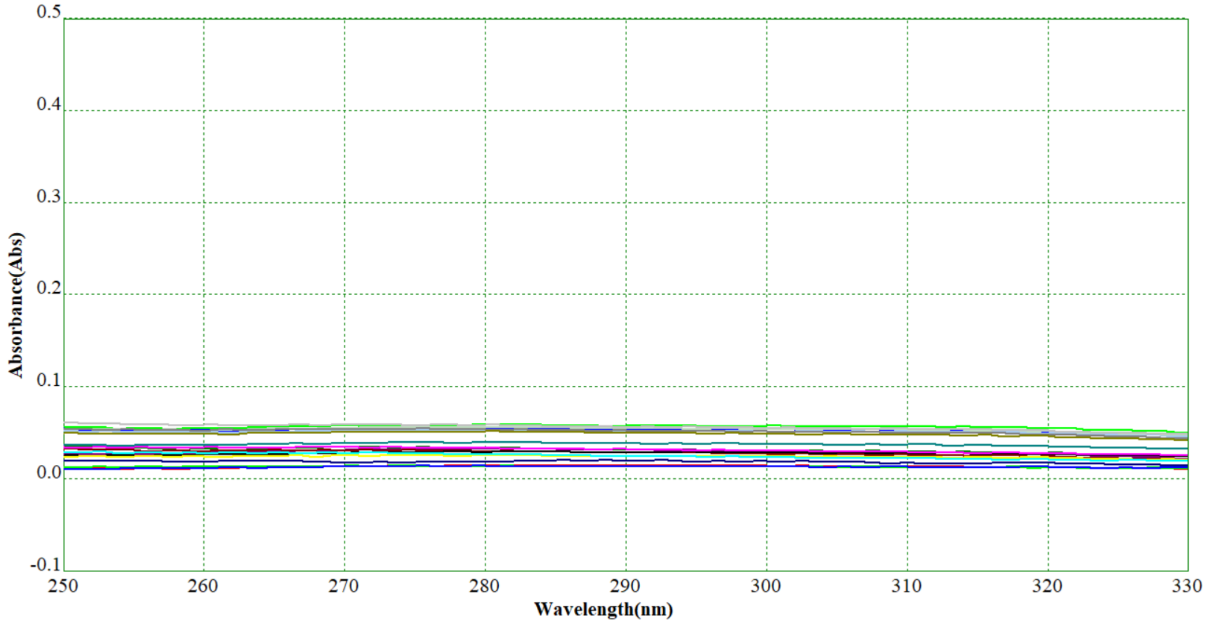

Figure S17. Superimposed UV-Vis spectra for the controlled release studies on the transdermal pharmaceutical formulation based on β-CD/*M. chamomilla* root extract complex in ethanol 20% solution; raw data obtained by monitoring the absorbance of the supernatant solution in the range of 250-400 nm for 90 min (a number of 19 spectra were recorded every 5 min, starting from 0 min – the lowest spectra to 90 min – the highest spectra).

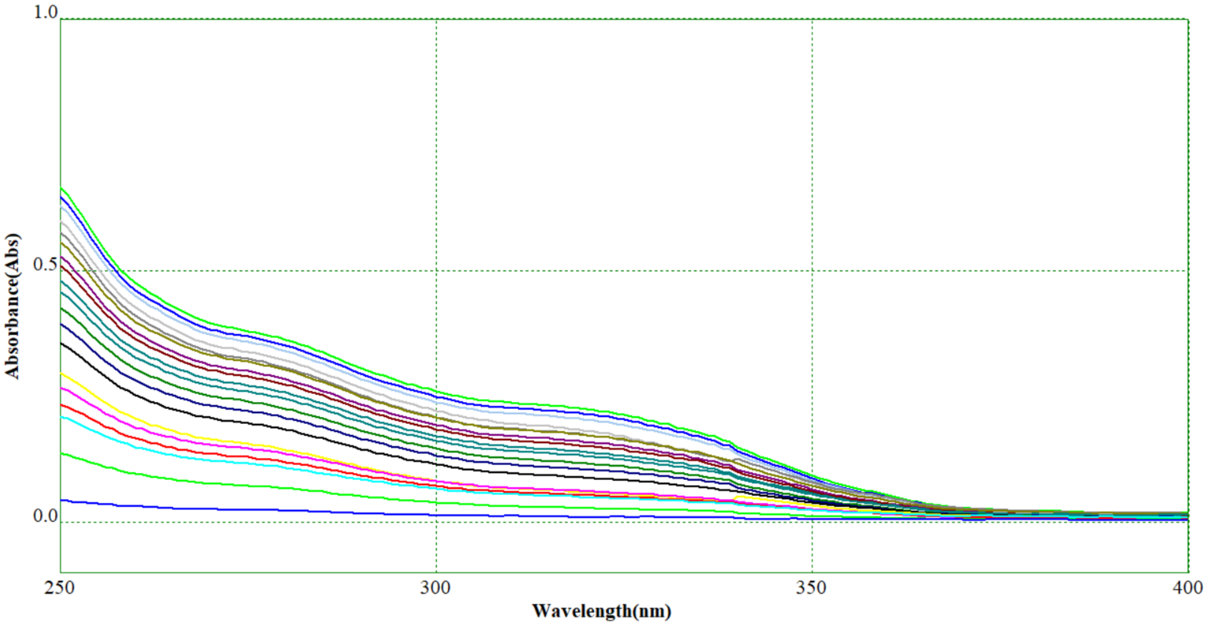

Figure S18. Superimposed UV-Vis spectra for the controlled release studies on the transdermal pharmaceutical formulation based on β-CD/*M. chamomilla* root extract complex in ethanol 60% solution; raw data obtained by monitoring the absorbance of the supernatant solution in the range of 250-400 nm for 90 min (a number of 19 spectra were recorded every 5 min, starting from 0 min – the lowest spectra to 90 min – the highest spectra).

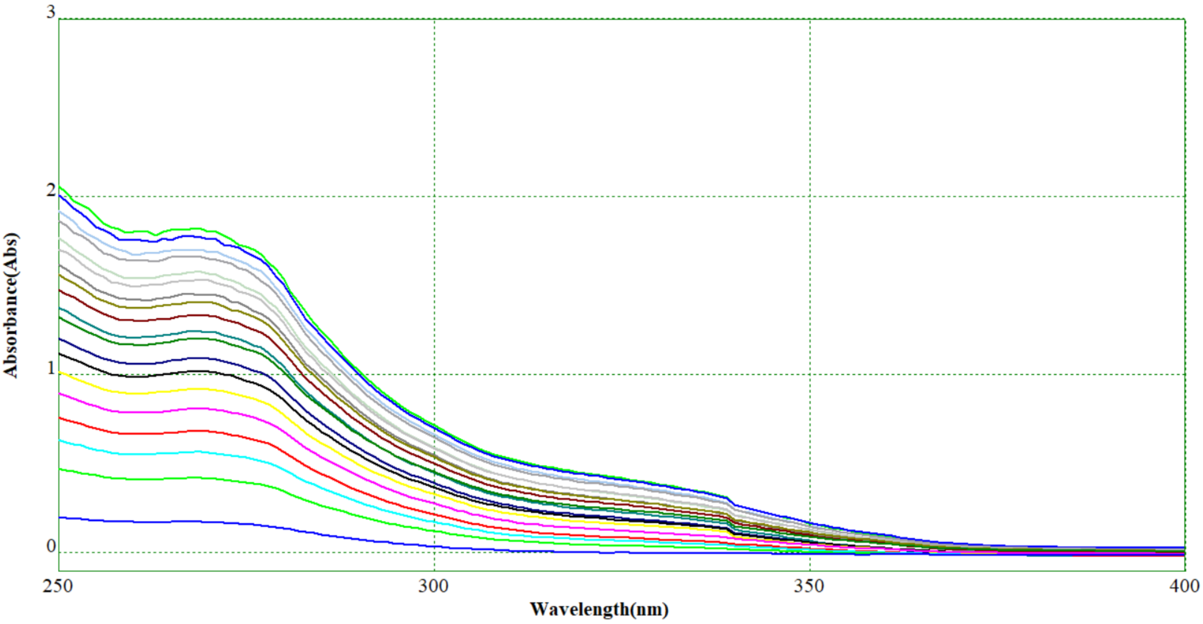

Figure S19. Superimposed UV-Vis spectra for the controlled release studies on the transdermal pharmaceutical formulation based on β-CD/*M. chamomilla* root extract complex in ethanol 96% solution; raw data obtained by monitoring the absorbance of the supernatant solution in the range of 250-400 nm for 90 min (a number of 19 spectra were recorded every 5 min, starting from 0 min – the lowest spectra to 90 min – the highest spectra).

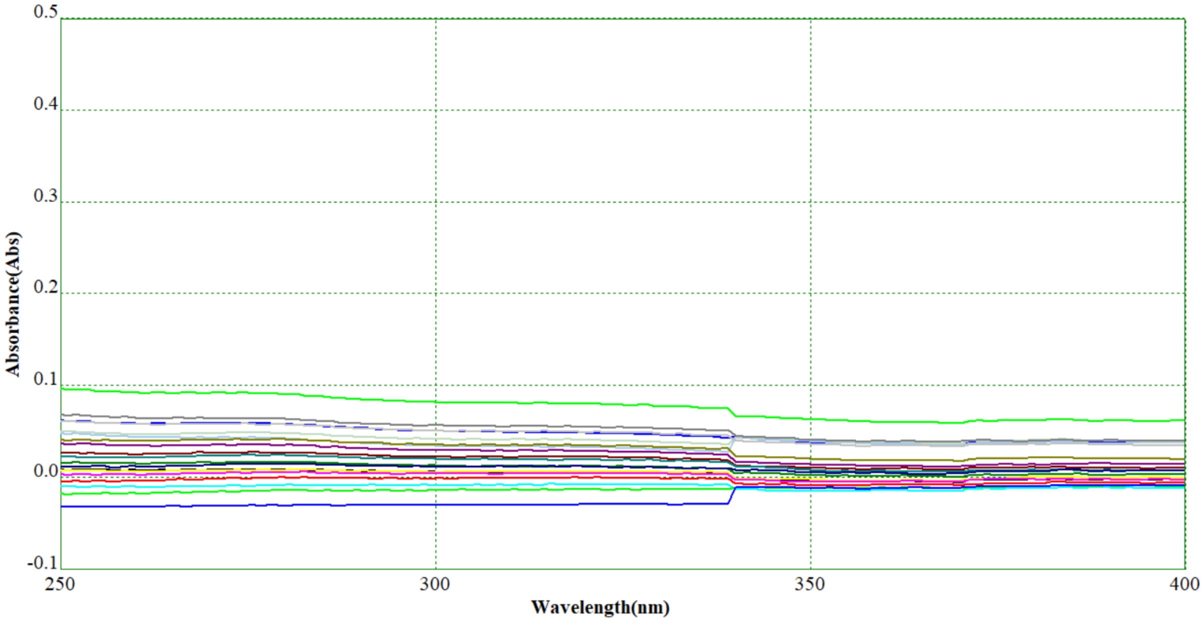

Figure S20. Superimposed UV-Vis spectra for the controlled release studies on the transdermal pharmaceutical formulation based on β-CD/*M. chamomilla* stem extract complex in saline solution; raw data obtained by monitoring the absorbance of the supernatant solution in the range of 250-400 nm for 90 min (a number of 19 spectra were recorded every 5 min, starting from 0 min – the lowest spectra to 90 min – the highest spectra).

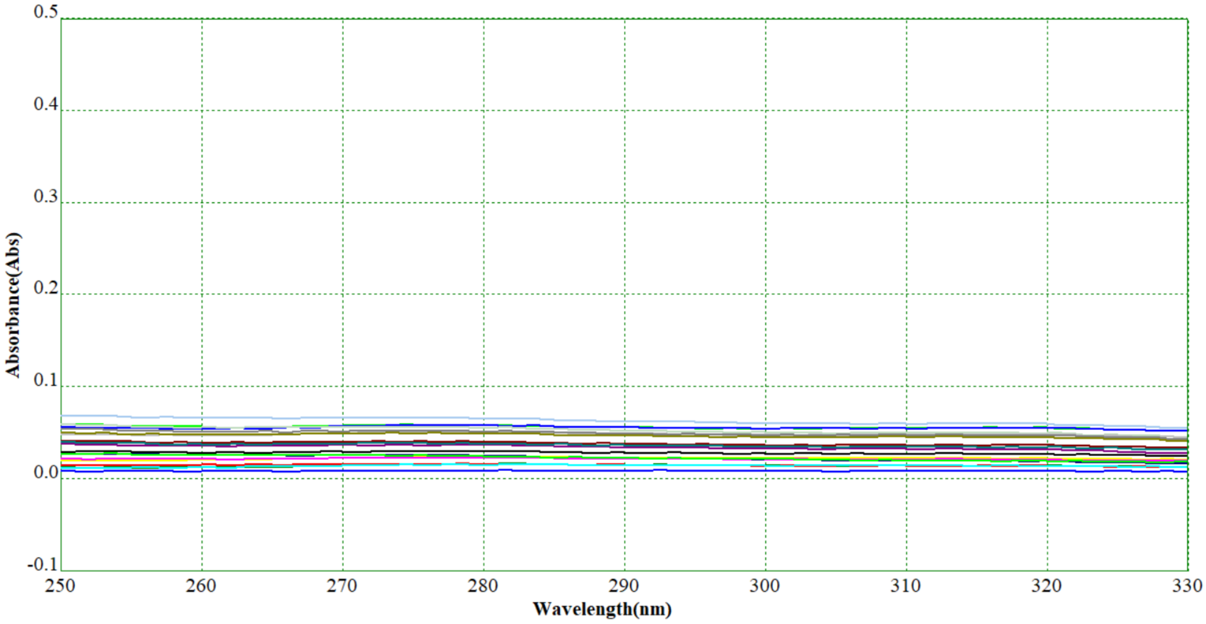

Figure S21. Superimposed UV-Vis spectra for the controlled release studies on the transdermal pharmaceutical formulation based on β-CD/*M. chamomilla* stem extract complex in ethanol 20% solution; raw data obtained by monitoring the absorbance of the supernatant solution in the range of 250-400 nm for 90 min (a number of 19 spectra were recorded every 5 min, starting from 0 min – the lowest spectra to 90 min – the highest spectra).

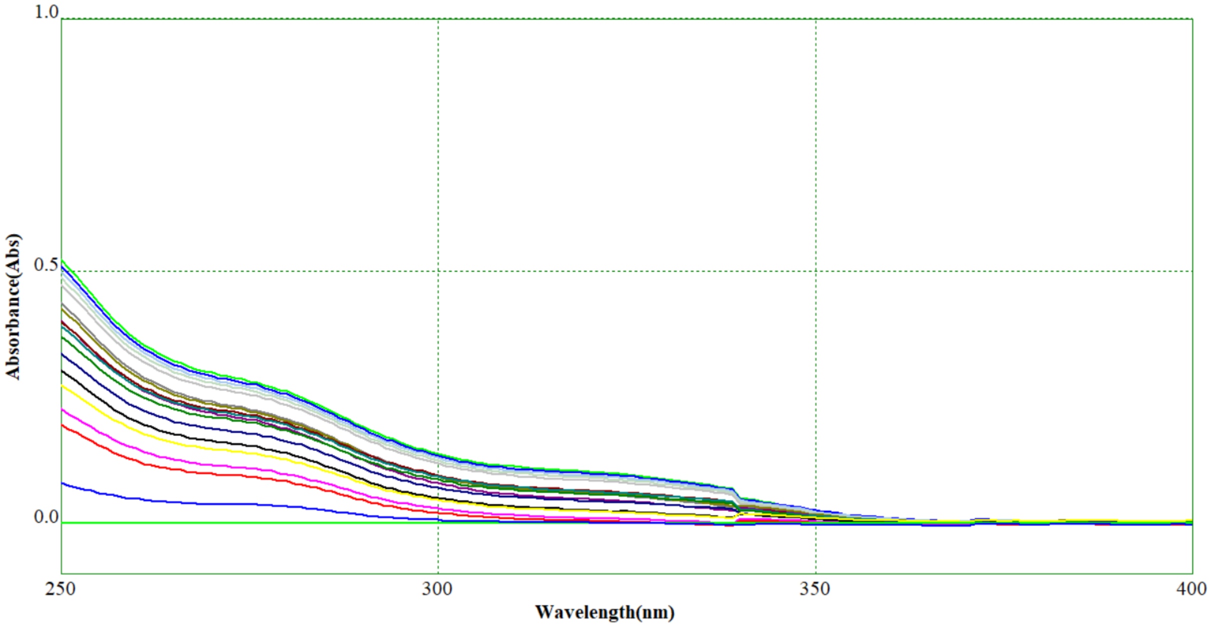

Figure S22. Superimposed UV-Vis spectra for the controlled release studies on the transdermal pharmaceutical formulation based on β-CD/*M. chamomilla* stem extract complex in ethanol 60% solution; raw data obtained by monitoring the absorbance of the supernatant solution in the range of 250-400 nm for 90 min (a number of 19 spectra were recorded every 5 min, starting from 0 min – the lowest spectra to 90 min – the highest spectra).

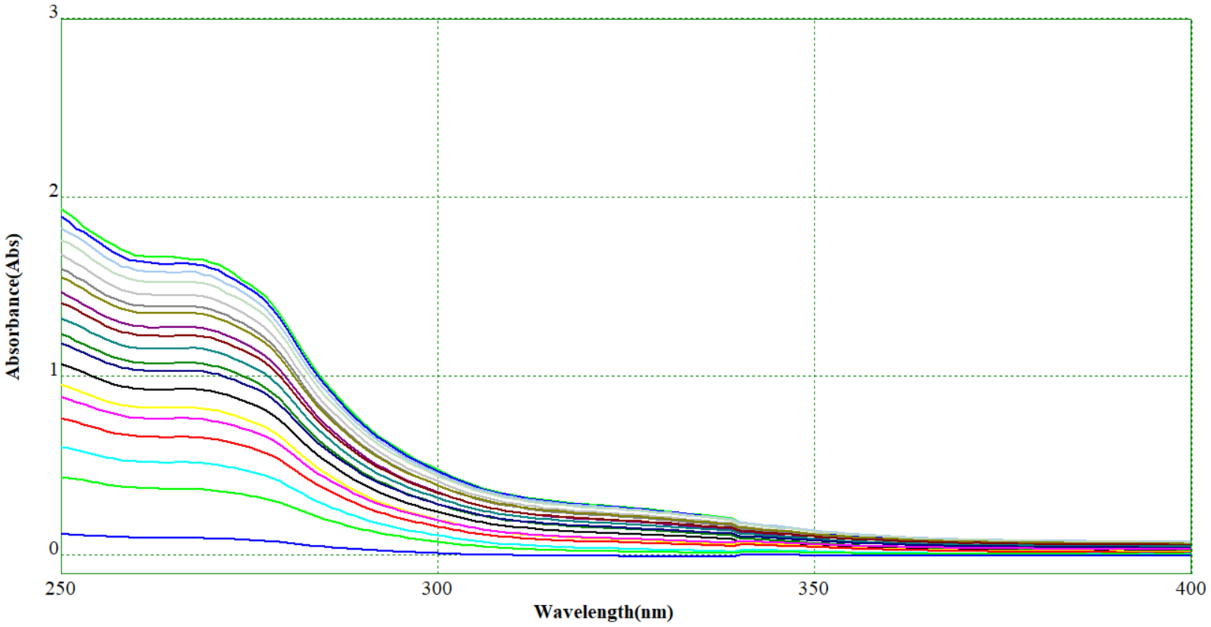

Figure S23. Superimposed UV-Vis spectra for the controlled release studies on the transdermal pharmaceutical formulation based on β-CD/*M. chamomilla* stem extract complex in ethanol 96% solution; raw data obtained by monitoring the absorbance of the supernatant solution in the range of 250-400 nm for 90 min (a number of 19 spectra were recorded every 5 min, starting from 0 min – the lowest spectra to 90 min – the highest spectra).

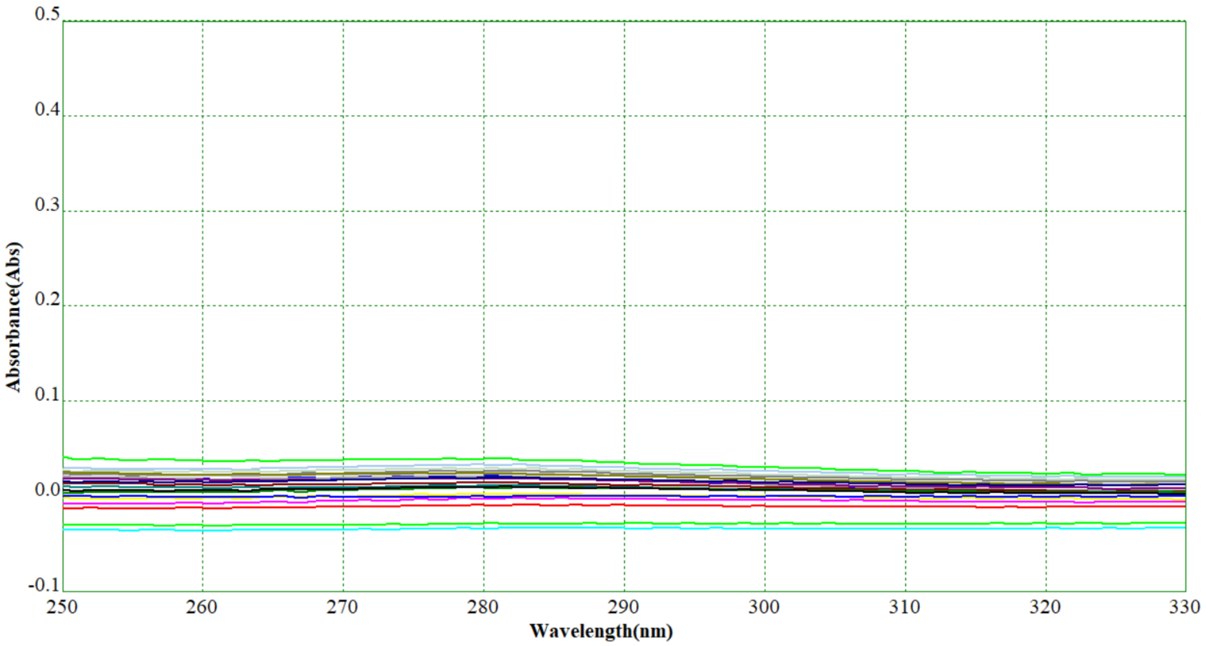

Figure S24. Superimposed UV-Vis spectra for the controlled release studies on the transdermal pharmaceutical formulation based on β-CD/*S. marianum* seed extract complex in saline solution; raw data obtained by monitoring the absorbance of the supernatant solution in the range of 250-400 nm for 90 min (a number of 19 spectra were recorded every 5 min, starting from 0 min – the lowest spectra to 90 min – the highest spectra).

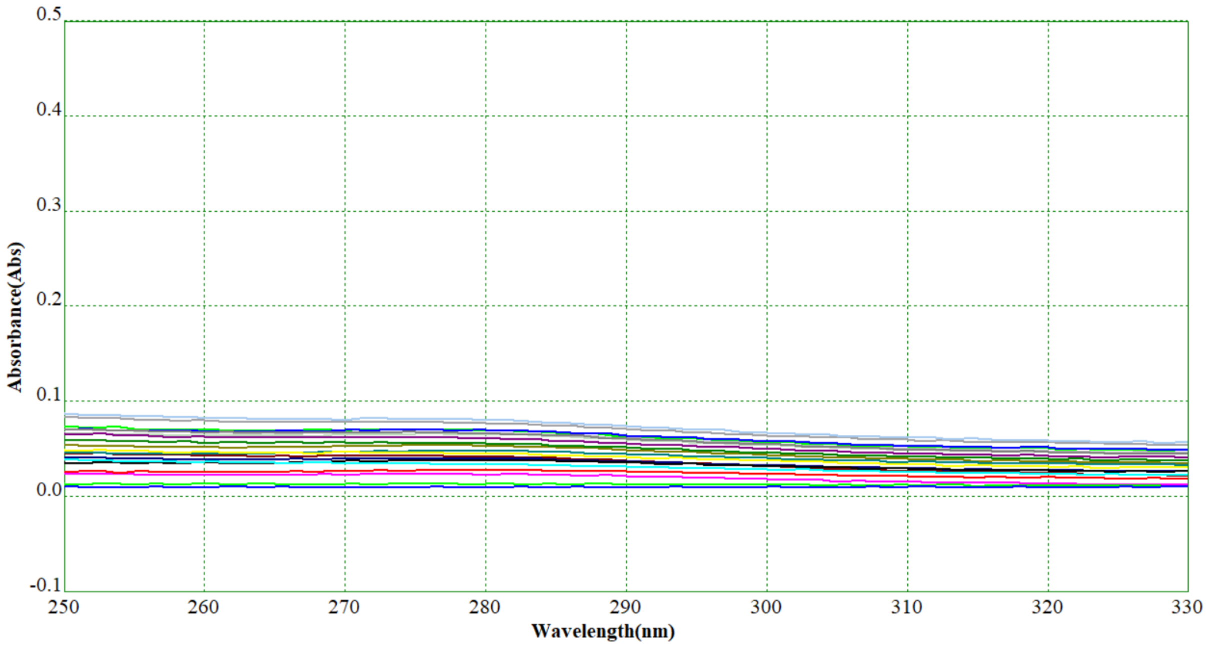

Figure S25. Superimposed UV-Vis spectra for the controlled release studies on the transdermal pharmaceutical formulation based on β-CD/*S. marianum* seed extract complex in ethanol 20% solution; raw data obtained by monitoring the absorbance of the supernatant solution in the range of 250-400 nm for 90 min (a number of 19 spectra were recorded every 5 min, starting from 0 min – the lowest spectra to 90 min – the highest spectra).

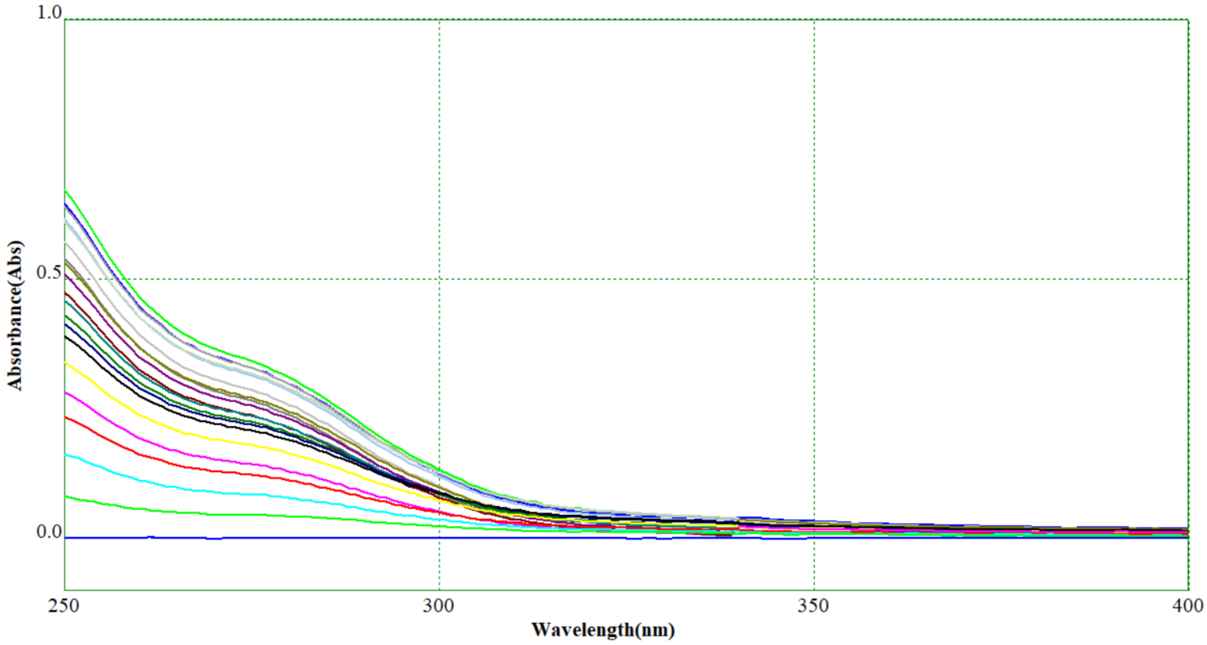

Figure S26. Superimposed UV-Vis spectra for the controlled release studies on the transdermal pharmaceutical formulation based on β-CD/*S. marianum* seed extract complex in ethanol 60% solution; raw data obtained by monitoring the absorbance of the supernatant solution in the range of 250-400 nm for 90 min (a number of 19 spectra were recorded every 5 min, starting from 0 min – the lowest spectra to 90 min – the highest spectra).

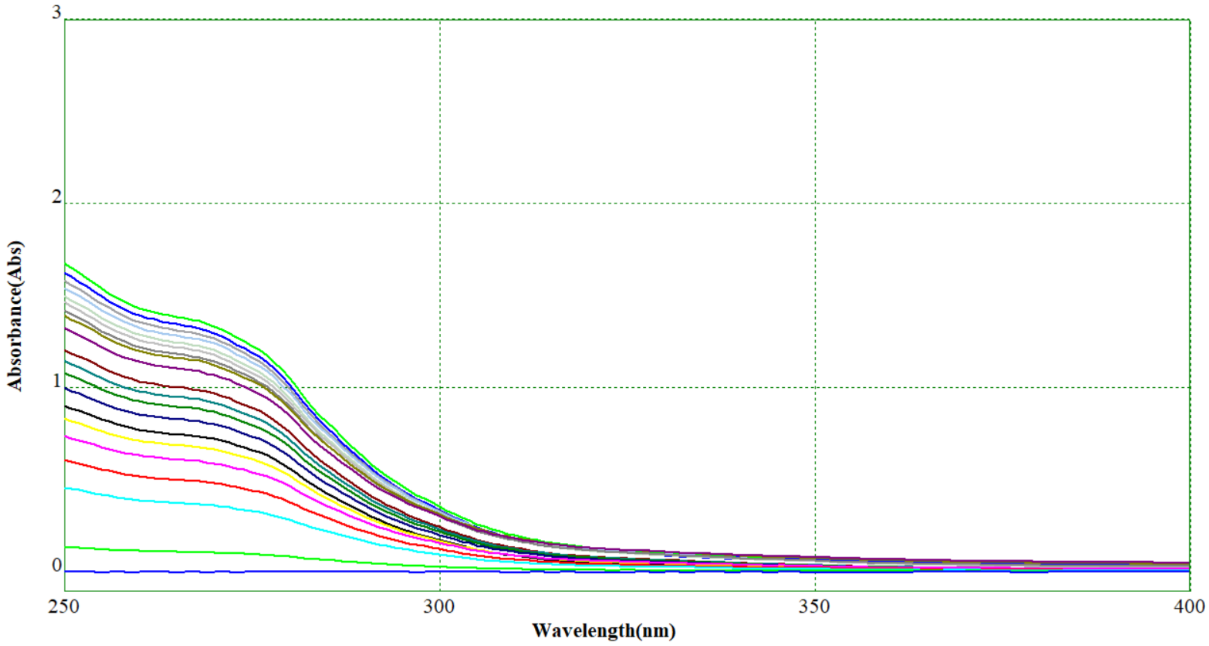

Figure S27. Superimposed UV-Vis spectra for the controlled release studies on the transdermal pharmaceutical formulation based on β-CD/*S. marianum* seed extract complex in ethanol 96% solution; raw data obtained by monitoring the absorbance of the supernatant solution in the range of 250-400 nm for 90 min (a number of 19 spectra were recorded every 5 min, starting from 0 min – the lowest spectra to 90 min – the highest spectra).

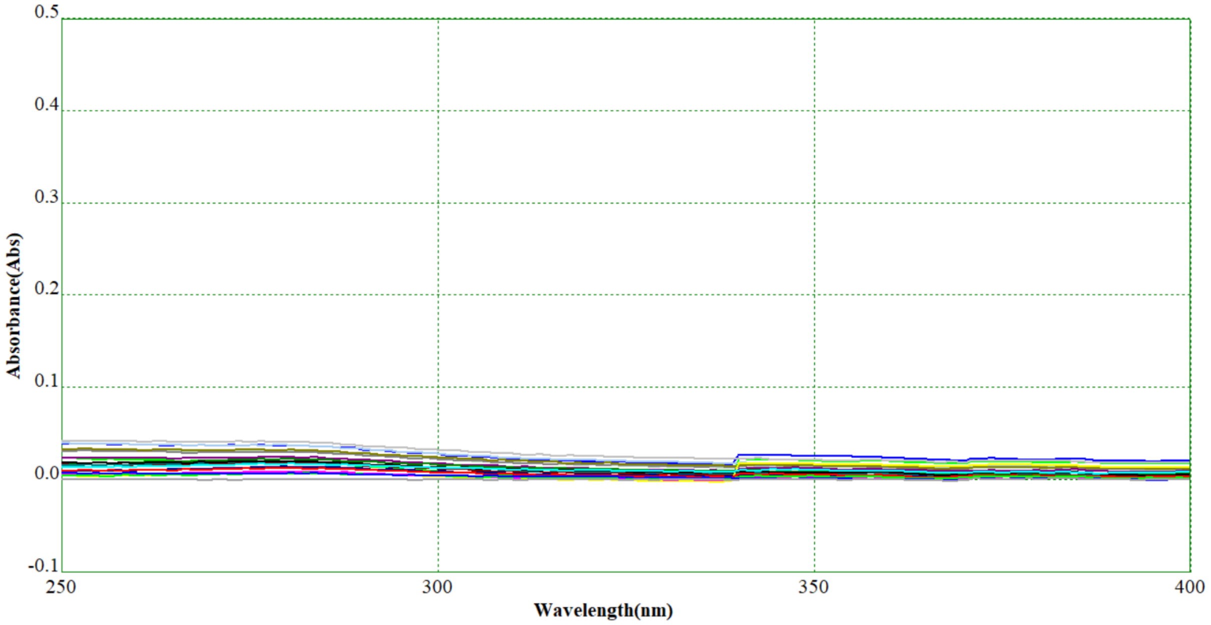

Figure S28. Superimposed UV-Vis spectra for the controlled release studies on the transdermal pharmaceutical formulation based on β-CD/silibinin diastereomer mixture complex in saline solution; raw data obtained by monitoring the absorbance of the supernatant solution in the range of 250-400 nm for 90 min (a number of 19 spectra were recorded every 5 min, starting from 0 min – the lowest spectra to 90 min – the highest spectra).

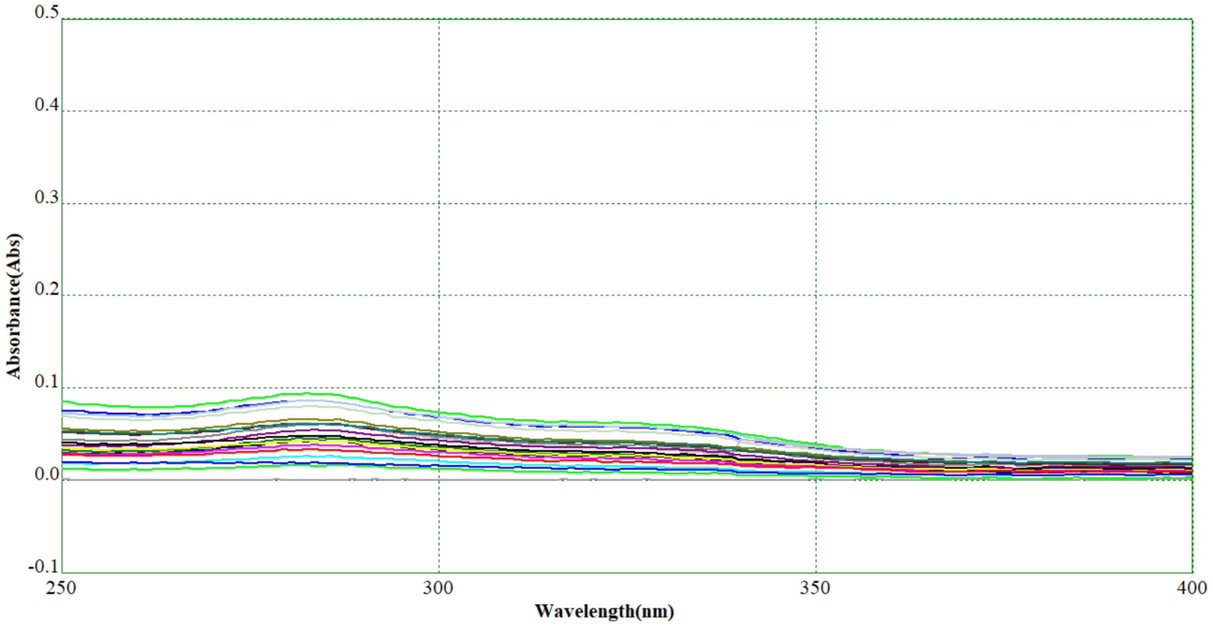

Figure S29. Superimposed UV-Vis spectra for the controlled release studies on the transdermal pharmaceutical formulation based on β-CD/silibinin diastereomer mixture complex in ethanol 20% solution; raw data obtained by monitoring the absorbance of the supernatant solution in the range of 250-400 nm for 90 min (a number of 19 spectra were recorded every 5 min, starting from 0 min – the lowest spectra to 90 min – the highest spectra).

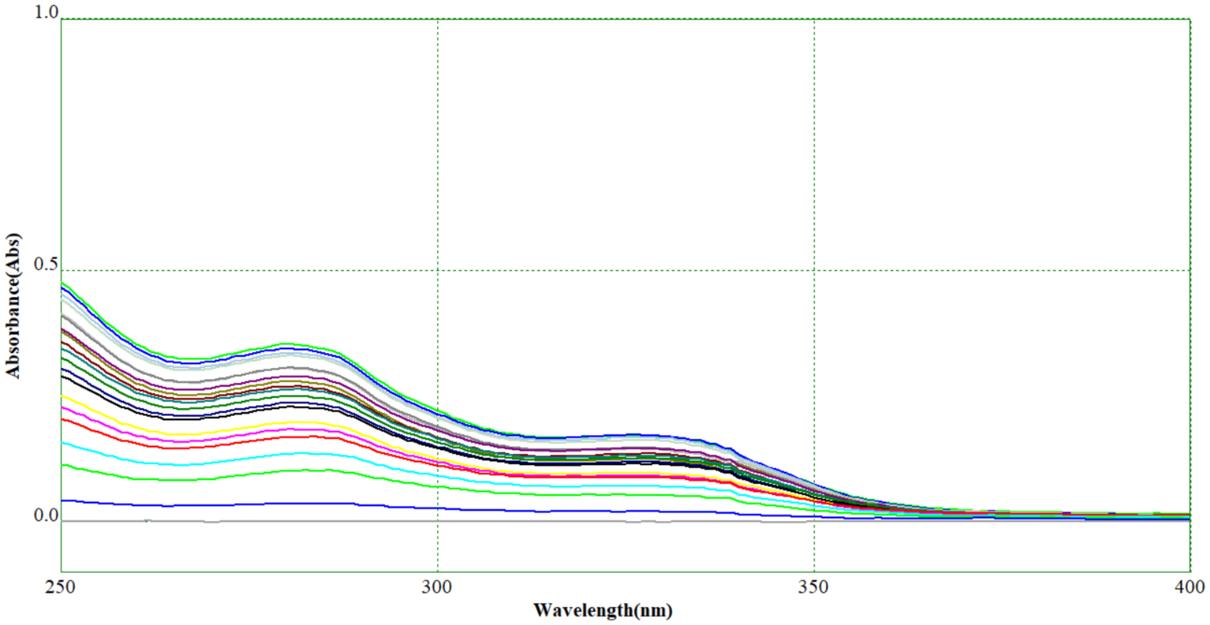

Figure S30. Superimposed UV-Vis spectra for the controlled release studies on the transdermal pharmaceutical formulation based on β-CD/silibinin diastereomer mixture complex in ethanol 60% solution; raw data obtained by monitoring the absorbance of the supernatant solution in the range of 250-400 nm for 90 min (a number of 19 spectra were recorded every 5 min, starting from 0 min – the lowest spectra to 90 min – the highest spectra).

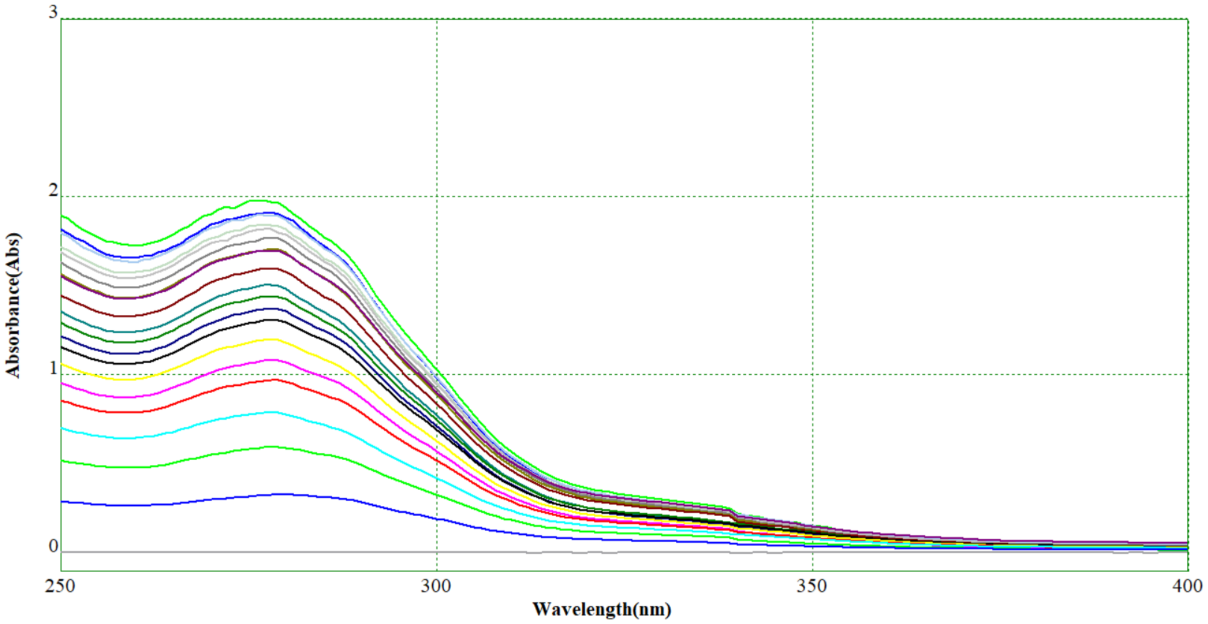

Figure S31. Superimposed UV-Vis spectra for the controlled release studies on the transdermal pharmaceutical formulation based on β-CD/silibinin diastereomer mixture complex in ethanol 96% solution; raw data obtained by monitoring the absorbance of the supernatant solution in the range of 250-400 nm for 90 min (a number of 19 spectra were recorded every 5 min, starting from 0 min – the lowest spectra to 90 min – the highest spectra).

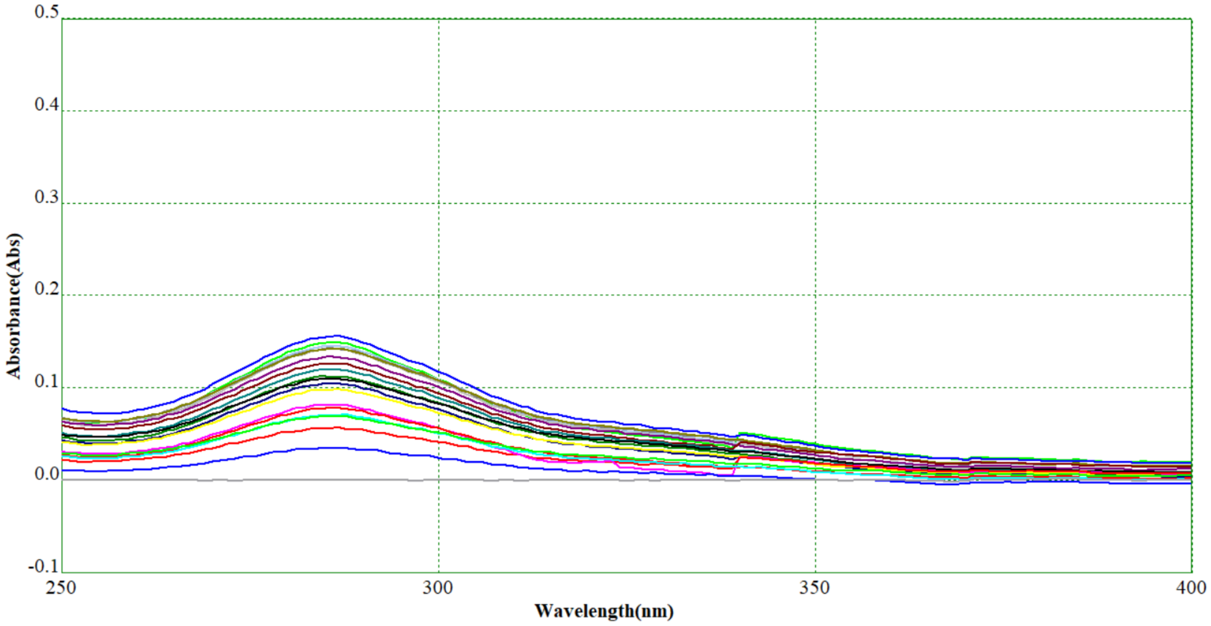

Figure S32. Superimposed UV-Vis spectra for the controlled release studies on the transdermal pharmaceutical formulation based on β-CD/silymarin mixture complex in saline solution; raw data obtained by monitoring the absorbance of the supernatant solution in the range of 250-400 nm for 90 min (a number of 19 spectra were recorded every 5 min, starting from 0 min – the lowest spectra to 90 min – the highest spectra).

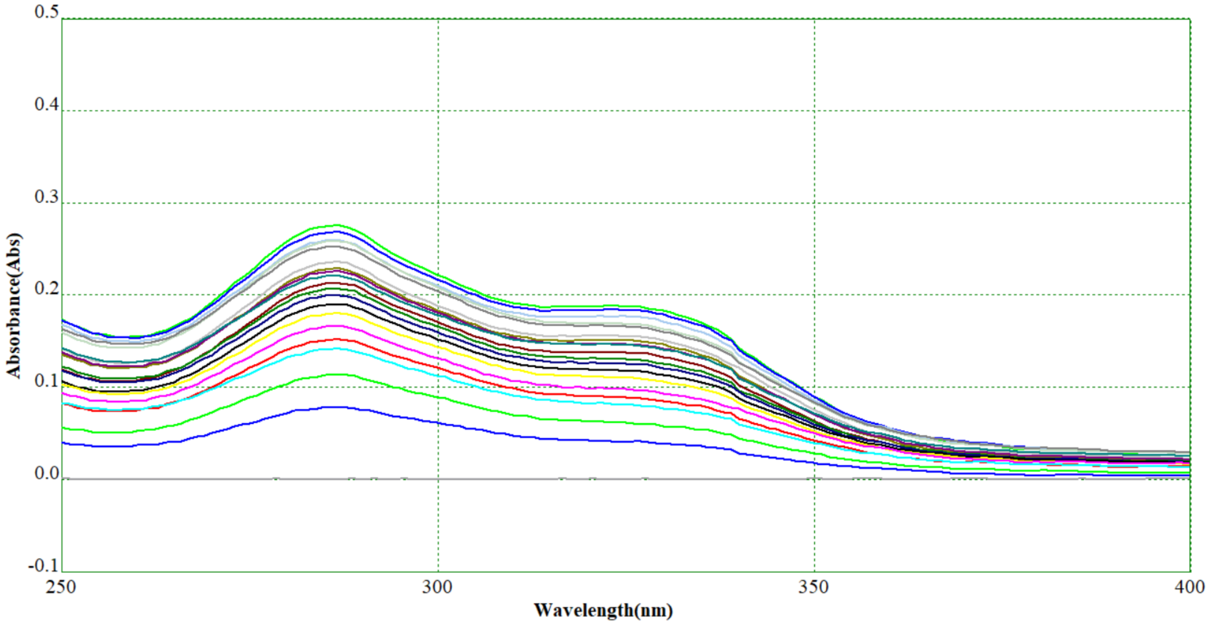

Figure S33. Superimposed UV-Vis spectra for the controlled release studies on the transdermal pharmaceutical formulation based on β-CD/silymarin mixture complex in ethanol 20% solution; raw data obtained by monitoring the absorbance of the supernatant solution in the range of 250-400 nm for 90 min (a number of 19 spectra were recorded every 5 min, starting from 0 min – the lowest spectra to 90 min – the highest spectra).

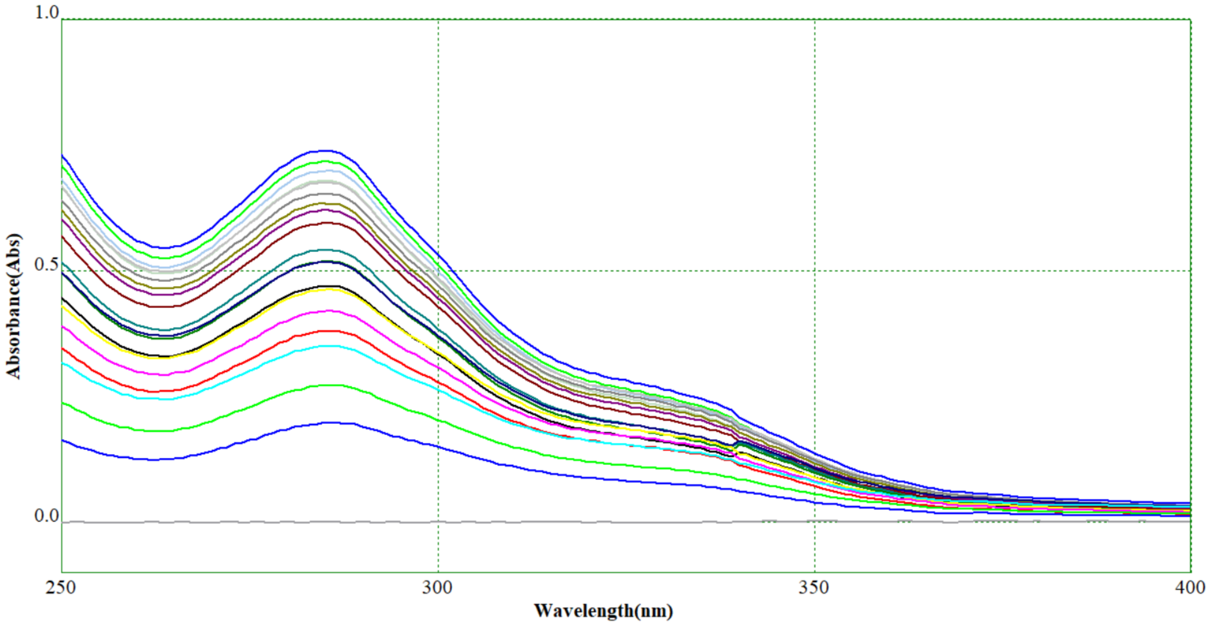

Figure S34. Superimposed UV-Vis spectra for the controlled release studies on the transdermal pharmaceutical formulation based on β-CD/silymarin mixture complex in ethanol 60% solution; raw data obtained by monitoring the absorbance of the supernatant solution in the range of 250-400 nm for 90 min (a number of 19 spectra were recorded every 5 min, starting from 0 min – the lowest spectra to 90 min – the highest spectra).

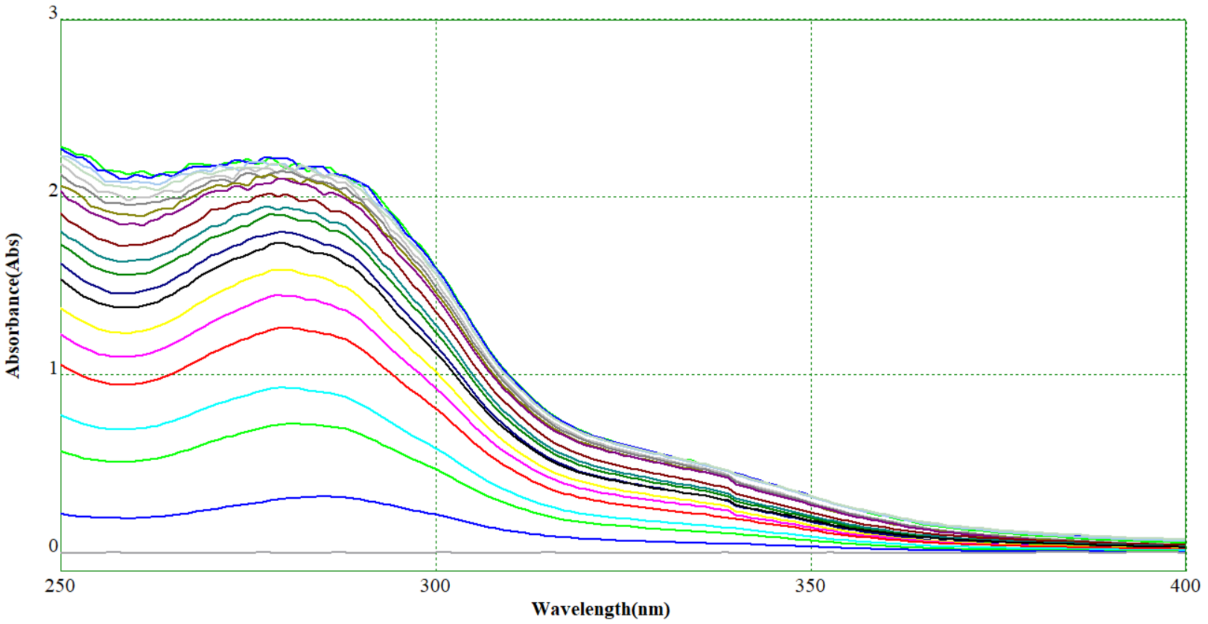

Figure S35. Superimposed UV-Vis spectra for the controlled release studies on the transdermal pharmaceutical formulation based on β-CD/silymarin mixture complex in ethanol 96% solution; raw data obtained by monitoring the absorbance of the supernatant solution in the range of 250-400 nm for 90 min (a number of 19 spectra were recorded every 5 min, starting from 0 min – the lowest spectra to 90 min – the highest spectra).

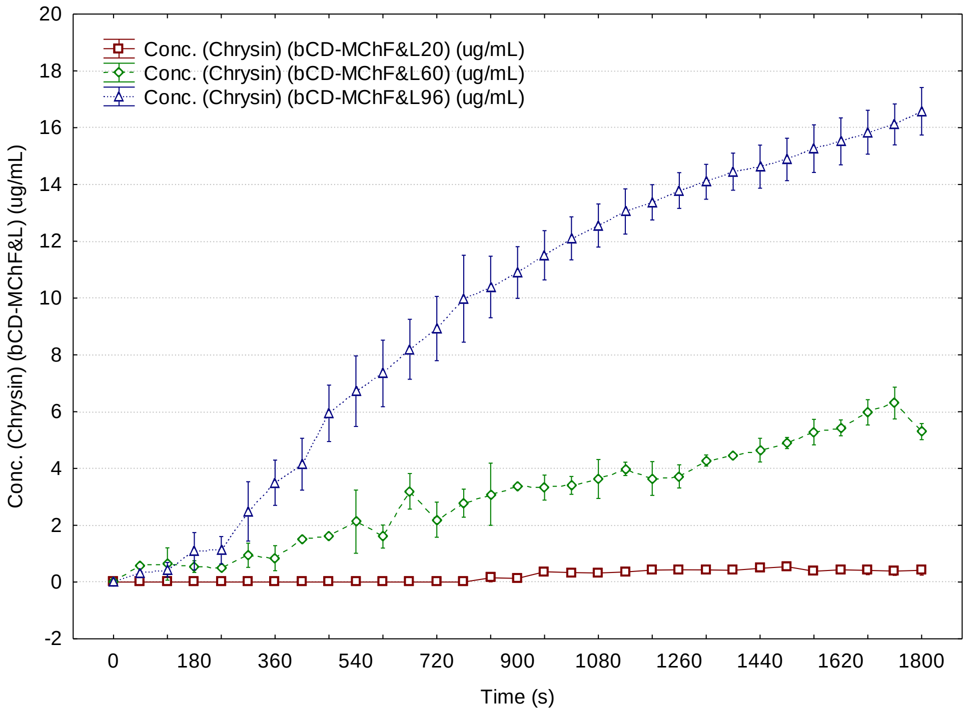

Figure S36. Controlled release of antioxidant compounds from the β-cyclodextrin/*M. chamomilla* flower and leaf extract complexes (expressed as chrysin, µg/mL) in ethanol 20% (brown), ethanol 60% (green) and ethanol 96% (bleu); number of replicate determinations *n* = 2; error bars were determined from standard errors using a coefficient of ±0.95.

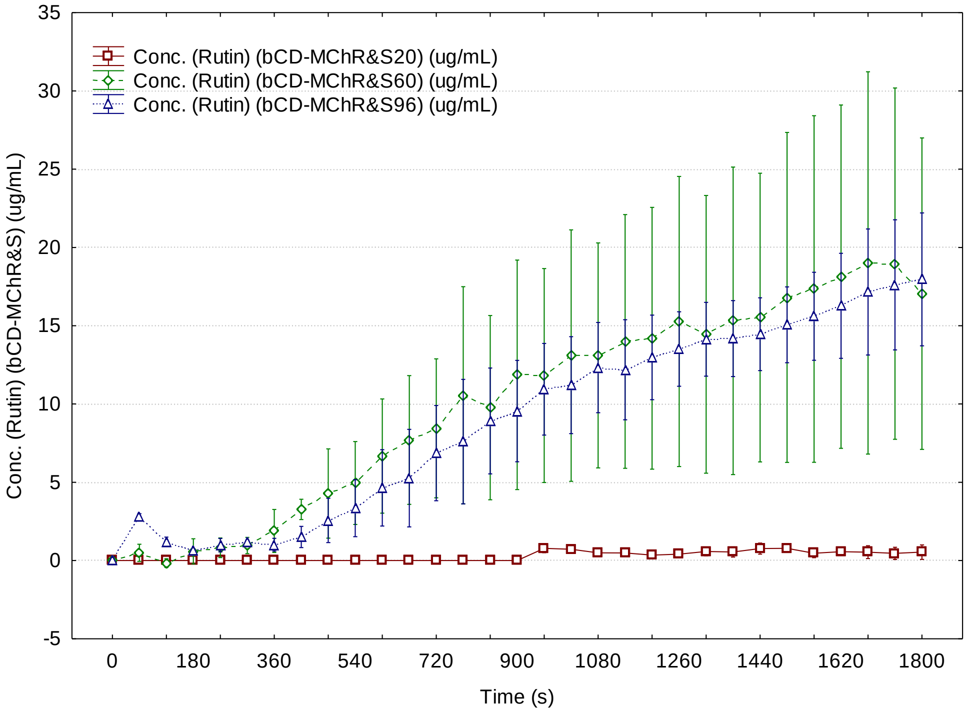

Figure S37. Controlled release of antioxidant compounds from the β-cyclodextrin/*M. chamomilla* root and stem extract complexes (expressed as rutin, µg/mL) in ethanol 20% (brown), ethanol 60% (green) and ethanol 96% (bleu); number of replicate determinations *n* = 2; error bars were determined from standard errors using a coefficient of ±0.95.

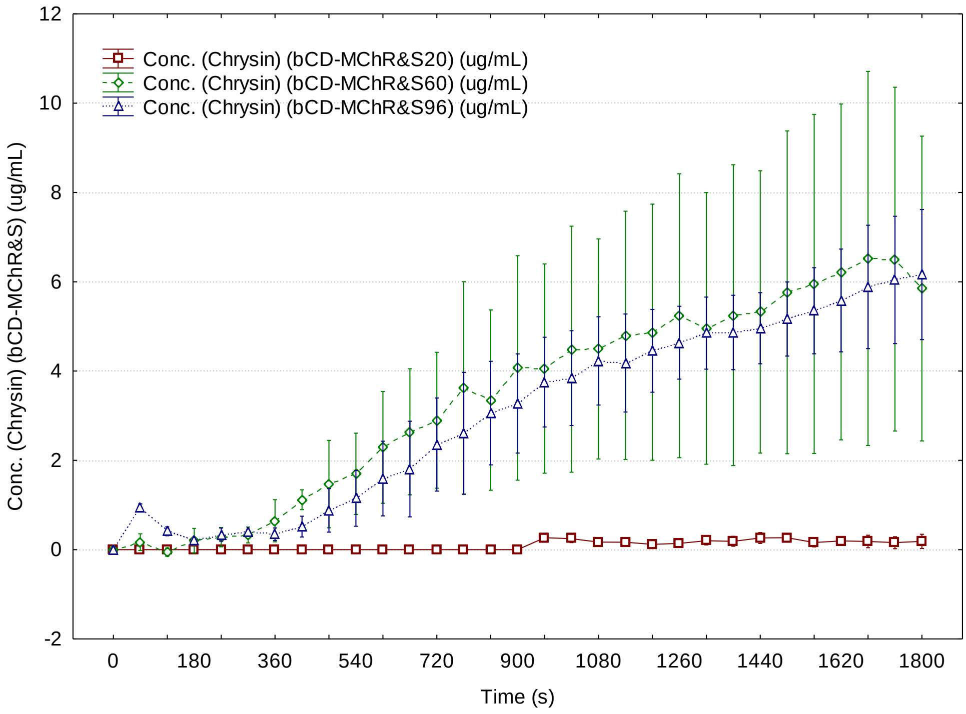

Figure S38. Controlled release of antioxidant compounds from the β-cyclodextrin/*M. chamomilla* root and stem extract complexes (expressed as chrysin, μg/mL) in ethanol 20% (brown), ethanol 60% (green) and ethanol 96% (bleu); number of replicate determinations *n* = 2; error bars were determined from standard errors using a coefficient of ±0.95.

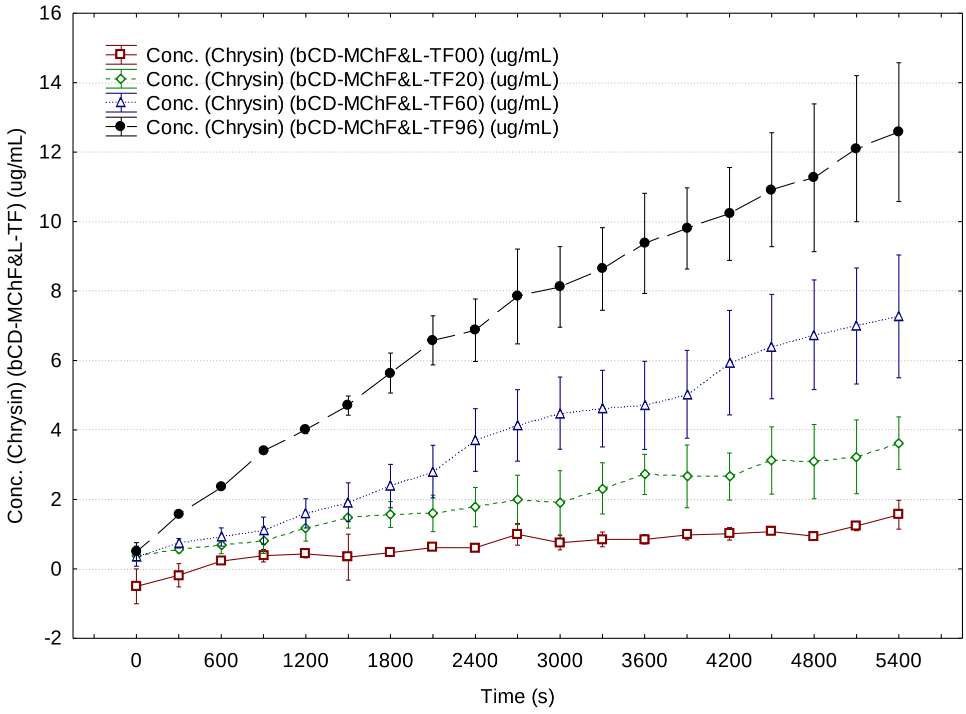

Figure S39. Controlled release of antioxidant compounds (expressed as chrysin, μg/mL) from the transdermal pharmaceutical formulations containing β-cyclodextrin/*M. chamomilla* flower and leaf extract complexes in saline solution (brown), ethanol 20% (green), ethanol 60% (bleu), and ethanol 96% (black); number of replicate determinations *n* = 2; error bars were determined from standard errors using a coefficient of ±0.95.

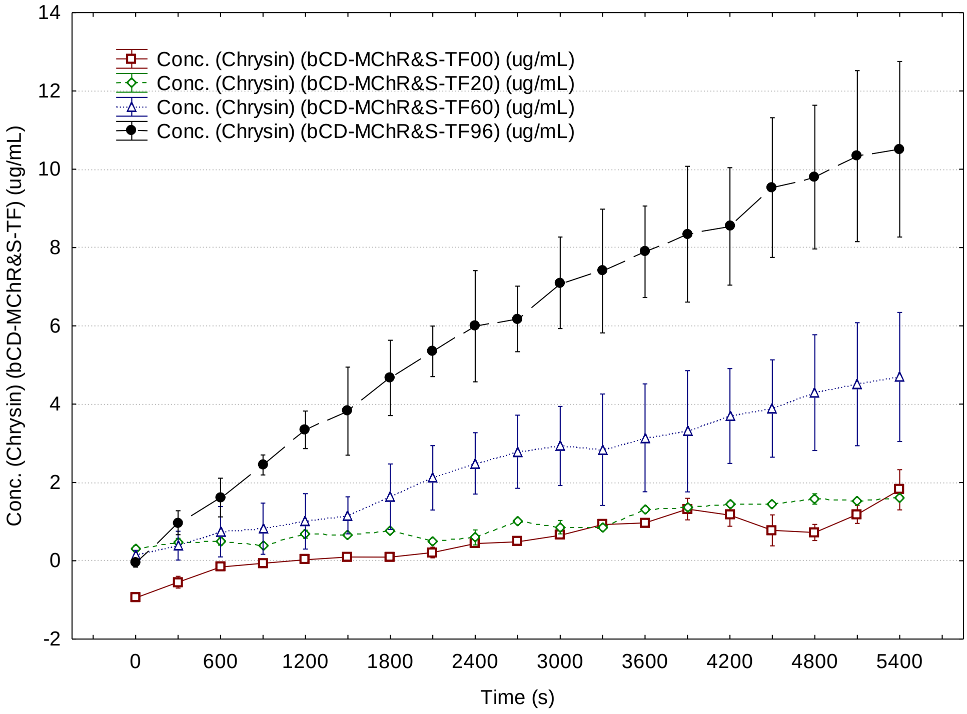

Figure S40. Controlled release of antioxidant compounds (expressed as chrysin, μg/mL) from the transdermal pharmaceutical formulations containing β-cyclodextrin/*M. chamomilla* root and stem extract complexes in saline solution (brown), ethanol 20% (green), ethanol 60% (bleu), and ethanol 96% (black); number of replicate determinations *n* = 2; error bars were determined from standard errors using a coefficient of ±0.95.

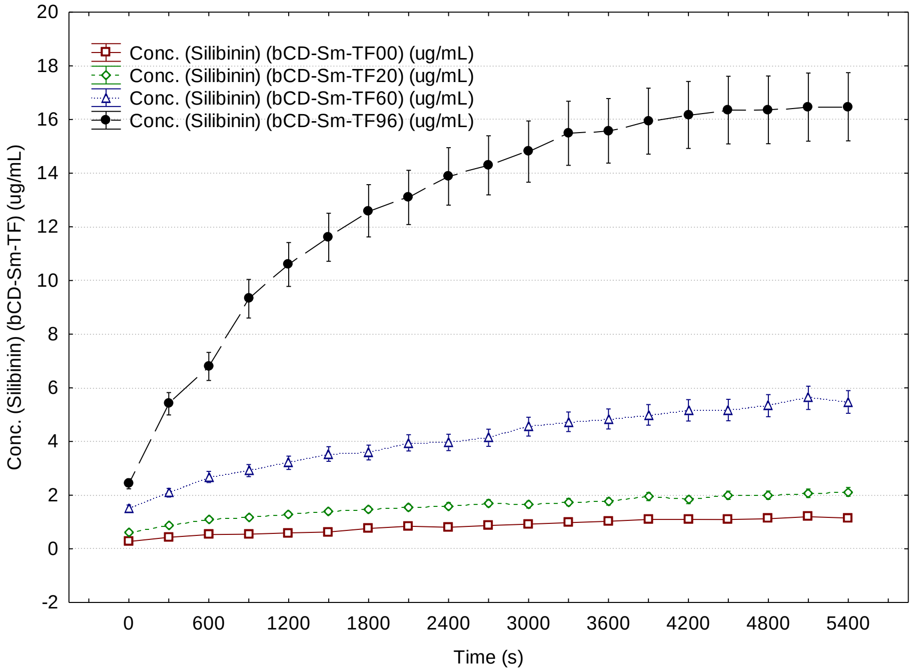

Figure S41. Controlled release of antioxidant compounds (expressed as silibinin, μg/mL) from the transdermal pharmaceutical formulations containing β-cyclodextrin/silymarin complexes in saline solution (brown), ethanol 20% (green), ethanol 60% (bleu), and ethanol 96% (black); number of replicate determinations *n* = 2; error bars were determined from standard errors using a coefficient of ±0.95.

Table S6. Korsmeyer-Peppas modeling ($M_t/M_\infty = k_{(KP)} \cdot t^n$) of the controlled release of antioxidants from β-cyclodextrin complexes. M_t and M_∞ stand for the amount (mg) of antioxidant released from the complex at the time *t* (s) and the total amount, respectively. The Korsmeyer-Peppas kinetic constant, $k_{(KP)}$ (s^{-n}), and the diffusional exponent, *n*, were determined by fitting the experimental data (least squares approximation). Codes for complexes are: *bCD_MChXY* – β-cyclodextrin/*M. chamomilla* L. extract; *bCD_SMaY* – β-cyclodextrin/*S. marianum* L. extract; *X* stands for the plant part, *X* = *F* – flowers, *L* – leaves, *R* – roots or *S* – stems; *bCD_Sb*/*SmY* – β-cyclodextrin/silibinin or β-cyclodextrin/silymarin complexes; *Y* stands for the concentration of ethanol solution used for controlled release (20, 60 and 96%, *v*/*v*).

Code	Korsmeyer-Peppas kinetic constant, $k_{(KP)}$ (s^{-n})	Diffusional exponent, *n*	Coefficient of determination, r^2
bCD_MChF20	$5.38 \cdot 10^{-7}$	1.314	0.769
bCD_MChF60	$1.05 \cdot 10^{-5}$	1.252	0.966
bCD_MChF96	$3.51 \cdot 10^{-4}$	0.922	0.985
bCD_MChL20	$1.13 \cdot 10^{-8}$	1.741	0.924
bCD_MChL60	$1.81 \cdot 10^{-4}$	0.760	0.967
bCD_MChL96	$5.86 \cdot 10^{-4}$	0.753	0.985
bCD_MChR20	$3.08 \cdot 10^{-4}$	0.261	0.670
bCD_MChR60	$6.13 \cdot 10^{-5}$	1.024	0.981
bCD_MChR96	$3.55 \cdot 10^{-5}$	1.042	0.978
bCD_MChS20	$1.7 \cdot 10^{-6}$	0.971	0.558
bCD_MChS60	$5.26 \cdot 10^{-5}$	0.942	0.966
bCD_MChS96	$5.17 \cdot 10^{-6}$	1.353	0.968
bCD_SMa20	$2.44 \cdot 10^{-5}$	0.808	0.904
bCD_SMa60	$7.45 \cdot 10^{-5}$	0.886	0.984
bCD_SMa96	$6.15 \cdot 10^{-4}$	0.656	0.960
bCD_Sb20	$1.29 \cdot 10^{-4}$	0.425	0.979
bCD_Sb60	$1.58 \cdot 10^{-5}$	0.935	0.993
bCD_Sb96	$3.81 \cdot 10^{-4}$	0.758	0.998
bCD_Sm20	$33.10 \cdot 10^{-4}$	0.320	0.893
bCD_Sm60	$6.84 \cdot 10^{-4}$	1.013	0.991
bCD_Sm96	$106.23 \cdot 10^{-4}$	0.371	0.975

Table S7. Korsmeyer-Peppas modeling ($M_t/M_\infty = k_{(KP)} \cdot t^n$) of the controlled release of antioxidants from transdermal pharmaceutical formulations based on β-cyclodextrin complexes. M_t and M_∞ stand for the amount (mg) of antioxidant released from the transdermal pharmaceutical formulation at the time *t* (s) and the total amount, respectively. The Korsmeyer-Peppas kinetic constant, $k_{(KP)}$ (s^{-n}), and the diffusional exponent, *n*, were determined by fitting the experimental data (least squares approximation). Codes for the transdermal pharmaceutical formulations based on cyclodextrin complexes are: *bCD_MChX_TFY* – transdermal pharmaceutical formulation based on β-cyclodextrin/*M. chamomilla* L. extract; *bCD_SMa_TFY* – transdermal pharmaceutical formulation based on β-cyclodextrin/*S. marianum* L. extract; *X* stands for the plant part, *X* = *F* – flowers, *L* – leaves, *R* – roots or *S* – stems; *bCD_Sb*/*Sm_TFY* – transdermal pharmaceutical formulation based on β-cyclodextrin/silibinin or β-cyclodextrin/silymarin complexes; *Y* stands for saline solution ("00") or the concentration of ethanol solution used for controlled release (20, 60 and 96%, *v*/*v*).

Code	Korsmeyer-Peppas kinetic constant, $k_{(KP)}$ (s^{-n})	Diffusional exponent, *n*	Coefficient of determination, r^2
bCD_MChF00	$5.25 \cdot 10^{-4}$	0.242	0.726
bCD_MChF20	$6.32 \cdot 10^{-4}$	0.342	0.823
bCD_MChF60	$6.36 \cdot 10^{-4}$	1.004	0.993
bCD_MChF96	$2.64 \cdot 10^{-4}$	0.641	0.996
bCD_MChL00	$2.60 \cdot 10^{-4}$	0.299	0.926
bCD_MChL20	$21.36 \cdot 10^{-4}$	0.178	0.810
bCD_MChL60	$1.21 \cdot 10^{-4}$	0.612	0.973
bCD_MChL96	$1.06 \cdot 10^{-4}$	0.692	0.996
bCD_MChR00	$1.03 \cdot 10^{-4}$	0.335	0.911
bCD_MChR20	$4.22 \cdot 10^{-4}$	0.265	0.752
bCD_MChR60	$1.78 \cdot 10^{-4}$	0.544	0.976
bCD_MChR96	$1.49 \cdot 10^{-4}$	0.657	0.995
bCD_MChS00	$1.56 \cdot 10^{-4}$	0.471	0.925
bCD_MChS20	$20.49 \cdot 10^{-4}$	0.175	0.690
bCD_MChS60	$4.62 \cdot 10^{-5}$	0.703	0.959
bCD_MChS96	$9.46 \cdot 10^{-5}$	0.757	0.994
bCD_SMa00	$1.61 \cdot 10^{-4}$	0.137	0.791
bCD_SMa20	$6.03 \cdot 10^{-4}$	0.160	0.777
bCD_SMa60	$23.54 \cdot 10^{-4}$	0.145	0.824
bCD_SMa96	$8.29 \cdot 10^{-4}$	0.408	0.990
bCD_Sb00	$2.84 \cdot 10^{-5}$	0.022	0.367
bCD_Sb20	$7.65 \cdot 10^{-5}$	0.022	0.437
bCD_Sb60	$3.35 \cdot 10^{-4}$	0.022	0.639
bCD_Sb96	$2.81 \cdot 10^{-4}$	0.259	0.949
bCD_Sm00	$2.53 \cdot 10^{-4}$	0.022	0.564
bCD_Sm20	$4.71 \cdot 10^{-4}$	0.022	0.617
bCD_Sm60	$5.54 \cdot 10^{-4}$	0.118	0.786
bCD_Sm96	$15.76 \cdot 10^{-4}$	0.140	0.875
